# Calibration-Aware Heterogeneous Multimodal Ensemble for Multi-Class Skin Lesion Diagnosis

**DOI:** 10.3390/diagnostics16213613

**Published:** 2026-11-01

**Authors:** Ozan Alptekin, Feristah Dalkilic, Zerrin Isik

**Affiliations:** 1 Graduate School of Natural and Applied Sciences, Dokuz Eylul University, 35390 İzmir, Türkiye; ozan.alptekin@ogr.deu.edu.tr; 2 Department of Computer Engineering, Dokuz Eylul University, 35390 İzmir, Türkiye; feristah@cs.deu.edu.tr; 3 Bioinformatics Group, Department of Computer Science, Vrije Universiteit Amsterdam, 1081 HV Amsterdam, The Netherlands

**Keywords:** skin lesion classification, melanoma diagnosis, hybrid deep learning, transformer models, multimodal fusion, probability calibration

## Abstract

**Background:** Reliable classification of skin lesions is essential for early melanoma detection; however, substantial visual overlap among lesion types and pronounced class imbalance continue to limit the performance and reliability of existing automated systems. These challenges motivate the integration of complementary visual representations with clinical metadata to improve discrimination across lesion classes. **Methods:** This study introduces a calibrated multimodal ensemble learning framework for skin lesion classification. The proposed framework combines visual features extracted from Swin Transformer, EVA-02, and CoAtNet architectures with patient age and anatomical region information. A two-stage adaptive temperature scaling is applied to calibrate the predicted probabilities of the ensemble. **Results:** Experimental evaluation on the International Skin Imaging Collaboration (ISIC 2019) dataset demonstrates strong classification performance, achieving a macro-F1 score of 0.91 and a macro-AUC of 0.99. The framework further showed robust generalization on the official ISIC 2019 benchmark set, attaining a balanced accuracy of 0.65. The external validation on the PH^2^ dataset yielded a macro-F1 score of 0.93 and an ROC-AUC of 0.96. **Conclusions:** These findings demonstrate that combining heterogeneous visual representations, clinical metadata, and calibration-aware ensemble learning provides a robust and trustworthy solution for skin lesion classification and computer-aided dermatological diagnosis.

## 1. Introduction

Skin cancer represents one of the most commonly diagnosed malignancies worldwide and ranks fifth among all cancers according to the GLOBOCAN 2022 estimates [1]. In that year alone, non-melanoma skin cancers accounted for approximately 1.23 million new cases and more than 63,000 deaths, whereas cutaneous melanoma contributed an additional 331,000 cases and 57,000 deaths globally [2]. Recent epidemiological studies suggest that over one million new skin cancer cases are reported each year, the vast majority being non-melanoma types such as basal cell carcinoma (BCC) and squamous cell carcinoma (SCC), which occur nearly 18 to 20 times more often than melanoma [3]. Prolonged ultraviolet exposure, fair skin phototypes, genetic predisposition, multiple dysplastic nevi, and immunosuppression remain the most recognized etiological factors contributing to the global incidence of cutaneous malignancies [4,5]. Early large-scale studies showed that deep neural networks could achieve performance comparable to that of dermatologists in the classification of skin malignancies [6].

Early detection of melanoma greatly improves survival outcomes; yet, diagnosis based solely on dermatoscopic images remains a demanding task that depends on clinician expertise and is prone to observer variability. Consequently, computer-aided skin-lesion analysis has emerged as a vital research direction aimed at assisting dermatologists and streamlining diagnostic workflows. The earliest computer-aided diagnosis systems relied on conventional image-processing techniques (e.g., color and texture descriptors, lesion-border detection, and handcrafted feature extraction) which were then coupled with traditional machine-learning classifiers such as Support Vector Machines (SVM) and Random Forests [7]. With the rapid rise of deep learning, Convolutional Neural Networks (CNNs), and more recently Vision Transformers (ViTs) [8], the performance of automated lesion classification has improved substantially [9]. A substantial part of this progress can be attributed to the introduction of residual learning frameworks, which enabled the effective training of deeper and more accurate neural networks [10].

Despite the advances in computer-aided skin-lesion analysis, several challenges still hinder the routine clinical use of automated systems. In practice, many lesion categories share highly similar visual characteristics, particularly melanoma and benign nevi, making reliable discrimination difficult even for advanced models. This challenge is further compounded by the pronounced imbalance in available datasets, where rare but clinically important lesion types remain under-represented. Variations in imaging devices, acquisition settings, and patient demographics introduce additional sources of variability that affect model generalization. Moreover, deep learning models often produce poorly calibrated probability estimates, which limits their trustworthiness in clinical decision-making, while differences in evaluation protocols can unintentionally lead to data leakage and overly optimistic performance reporting. Efforts to standardize evaluation protocols and account for these sources of variability have been a key focus of the International Skin Imaging Collaboration (ISIC), which has established widely adopted benchmarks for reliable model comparison [11].

To address these challenges, this study introduces a meta-aware hybrid deep learning framework that integrates visual representations extracted from three complementary backbones—Swin (shifted window) Transformer [12], EVA-02 (a next-generation Transformer-based visual representation model) [13], and CoAtNet (convolution and attention network) [14]—with age and anatomical region metadata to enrich clinical context. At the heart of the framework lies the RobustMLP pipeline, which jointly enhances classification accuracy and probability calibration through a two-stage adaptive temperature-scaling strategy.

## 2. Related Work

### 2.1. Classical Dermoscopic Studies and Early Computer Vision Methods

The foundations of automated skin lesion analysis are closely linked to early dermoscopic research, which documented the substantial visual heterogeneity of skin tumors. Clinical studies highlighted the diagnostic difficulty of distinguishing keratinizing lesions such as actinic keratosis (AK) and squamous cell carcinoma (SCC), where morphological boundaries are often subtle or ambiguous [15,16]. Motivated by these observations, early computer vision approaches relied on handcrafted descriptors (e.g., color asymmetry, texture patterns, and border irregularity) to approximate clinical reasoning [7]. These systems typically achieved moderate classification performance under controlled experimental settings but showed limited robustness when exposed to real-world variability. The subsequent release of large, curated dermoscopic datasets such as HAM10000 [17], BCN20000 [18], and the broader ISIC archives [19] enabled a shift toward data-driven learning and more systematic benchmarking.

### 2.2. CNN-Based and Transfer Learning Approaches

With the availability of large-scale datasets, CNN-based architectures rapidly became the dominant paradigm for automated skin lesion diagnosis. Ensembles of multi-resolution EfficientNets enriched with metadata demonstrated that class imbalance could be partially mitigated on ISIC 2019, reporting a balanced accuracy of approximately 0.63 [20]. Transfer learning approaches such as modified GoogleNet reported high overall accuracy values exceeding 0.94 on hold-out test sets [21], while MobileNetV2–LSTM hybrids achieved accuracies around 0.85 on the HAM10000 dataset [22]. Despite these improvements, CNN-based models frequently exhibited reduced sensitivity for underrepresented classes and struggled with visually overlapping lesion categories, limiting their reliability in clinically challenging cases.

### 2.3. Metadata-Aware and Multimodal Architectures

Given that dermatological diagnosis relies heavily on contextual information, several studies have explored the integration of clinical metadata. Metadata-driven fusion strategies achieved balanced accuracy values around 0.85 and macro-AUC scores close to 0.98 [23]. Similarly, EfficientNet-based multimodal frameworks incorporating patient metadata reported improved AUC values for melanoma detection [24], while TabNet-based fusion models enhanced the interpretability of non-visual features [25]. Although these advances underline the importance of clinical context, reported gains remain inconsistent across lesion types, and many approaches rely on dataset-specific fusion strategies that limit generalization.

### 2.4. Transformers, Hybrid Models, and Generative Approaches

More recently, ViTs and hybrid CNN–Transformer architectures have been introduced to capture global contextual dependencies in dermoscopic images [26]. Swin Transformer models reported high overall accuracy values exceeding 0.97, although balanced accuracy remained substantially lower due to class imbalance [27]. Hybrid convolution–attention architectures reported balanced performance across all eight ISIC 2019 classes, with macro-level scores approaching 0.92 [28], while lightweight attention-based networks focused on computational efficiency with accuracies near 0.91 [29].

In parallel, generative approaches (i.e., GAN-based data augmentation) have been employed to mitigate class imbalance, reporting accuracy levels around 0.93 [30]. EfficientNet-B6 combined with test-time augmentation and explainability techniques further improved prediction reliability, achieving accuracy values close to 0.97 [31]. Despite these promising results, many transformer-based and generative models primarily emphasize classification accuracy, while probability calibration, clinical reliability, and cross-center generalization remain largely underexplored.

### 2.5. Key Limitations in Existing Literature

Despite substantial progress in automated skin lesion analysis, several challenges continue to hinder clinical adoption. First, strong visual similarities between lesion categories, particularly melanoma versus benign nevi and actinic keratosis versus squamous cell carcinoma, remain a major source of diagnostic uncertainty. Second, severe class imbalance in commonly used benchmark datasets often reduces sensitivity for minority classes. Third, many existing approaches rely exclusively on image data and therefore fail to exploit clinically relevant contextual information such as patient age and anatomical location. In addition, modern deep learning models frequently produce poorly calibrated probability estimates, limiting the reliability of confidence scores for clinical decision support [32,33]. Representative studies addressing these challenges, together with their reported classification performance on ISIC-based datasets, are summarized in the Discussion section.

These limitations motivate the development of clinically aligned and rigorously validated frameworks that jointly address visual ambiguity, class imbalance, metadata integration, probability calibration, and reproducibility.

### 2.6. Main Contributions

To address the limitations identified above, this study offers the following contributions:

- A heterogeneous multimodal ensemble framework is developed for multi-class skin lesion diagnosis by integrating visual representations from three architecturally diverse backbones, Swin-B, EVA-02, and CoAtNet, with patient age and anatomical-region metadata.
- A backbone-aware classification strategy is employed. Swin-B and EVA-02 use RobustMLP classifiers, whereas CoAtNet uses a class-balanced, L2-regularized logistic regression. This design accommodates the different feature representations while incorporating complementary visual and metadata information.
- A two-stage temperature-scaling strategy is applied to calibrate the heterogeneous ensemble. Model-specific temperatures are selected before weighted probability fusion, followed by a second temperature-scaling step applied to the fused ensemble probabilities.
- A multi-faceted evaluation protocol is conducted using classification, discrimination, and calibration metrics, with 95% confidence intervals, external validation on PH^2^, and comparison with reported results of the official ISIC 2019 benchmark test set.

## 3. Materials and Methods

**Figure 1:**
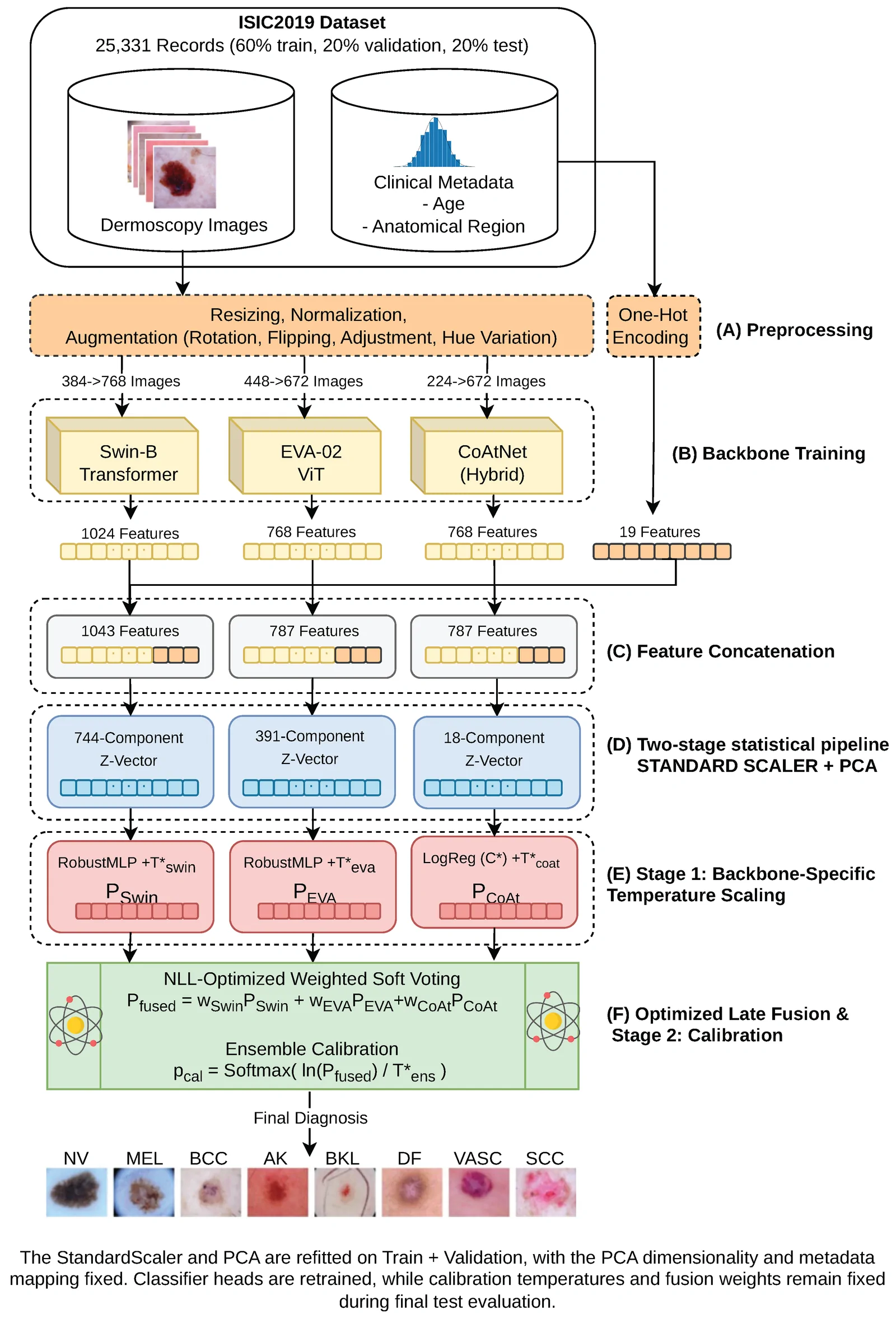
System overview of the proposed method. (**A**) Preprocessing of input images and metadata to align with models. (**B**) Training of the visual backbones using progressive resizing (384->768, 448->672, 224->672) (**C**) Concatenation of visual features and metadata features. (**D**) Standardizing the features with StandardScaler and then reducing the features via PCA to retain about 99% of the variance. (**E**) Backbone-specific classifiers and temperature scaling: RobustMLP for Swin-B and EVA-02, and class-weighted Logistic Regression for CoAtNet, followed by model-specific temperature scaling. (**F**) NLL-optimized weighted soft voting of calibrated base-model probabilities, followed by ensemble-level temperature scaling. Asterisks (*) denote values selected on the validation set: temperatures (T) chosen by minimizing negative log-likelihood and the logistic-regression inverse regularization strength (C) chosen by grid search.

The proposed methodology follows a structured multi-stage pipeline in which high-dimensional representations extracted from visual backbones, namely Swin-B Transformer, EVA-02 ViT, and CoAtNet, are enriched with patient-level metadata such as age and anatomical site. Dermoscopic images are first standardized through resizing, normalization, and diverse augmentation strategies, while metadata is encoded to ensure compatibility with the visual feature space. In this way, the model learns not only the morphological appearance of each lesion but also the clinical context that routinely guides expert diagnostic reasoning. As illustrated in Figure 1, the integrated framework produces its final predictions by combining backbone-specific classification, two-stage adaptive temperature scaling, and weighted soft voting.

### 3.1. Dataset

This study utilized the ISIC 2019 Challenge dataset [17], which contains 25,331 dermoscopic images acquired from multiple clinical centers and publicly available datasets (HAM10000, BCN20000, and MSK) together with patient metadata (age, anatomical region, and sex) [11,18]. The dataset comprises eight diagnostic categories: Melanoma (MEL), Melanocytic Nevus (NV), Basal Cell Carcinoma (BCC), Actinic Keratosis (AK), Benign Keratosis (BKL), Dermatofibroma (DF), Vascular Lesion (VASC), and Squamous Cell Carcinoma (SCC). Among these, differentiation between MEL and NV, as well as AK, BKL, MEL, and SCC, remains clinically challenging due to visual similarity and class imbalance [20]. This dataset was selected as the primary benchmark for its multi-source composition, which introduces substantial variability in image characteristics and acquisition conditions. This variability provides a challenging and representative setting for evaluating model robustness and generalizability. Furthermore, the availability of patient age and anatomical-region metadata directly supports the multimodal design of the proposed framework.

Images were resized to the native input resolution of each backbone and normalized. No additional color normalization or image enhancement was applied, preserving the original image characteristics and acquisition-related variability [19].

Patient age was discretized into nine decade-based groups and represented using a 9-dimensional one-hot encoding. Missing age values (1.7%) were encoded as all-zero vectors. Anatomical region information was encoded using a 10-category vocabulary that included the major body sites and a dedicated category for missing entries (10.4%).

**Figure 2:**
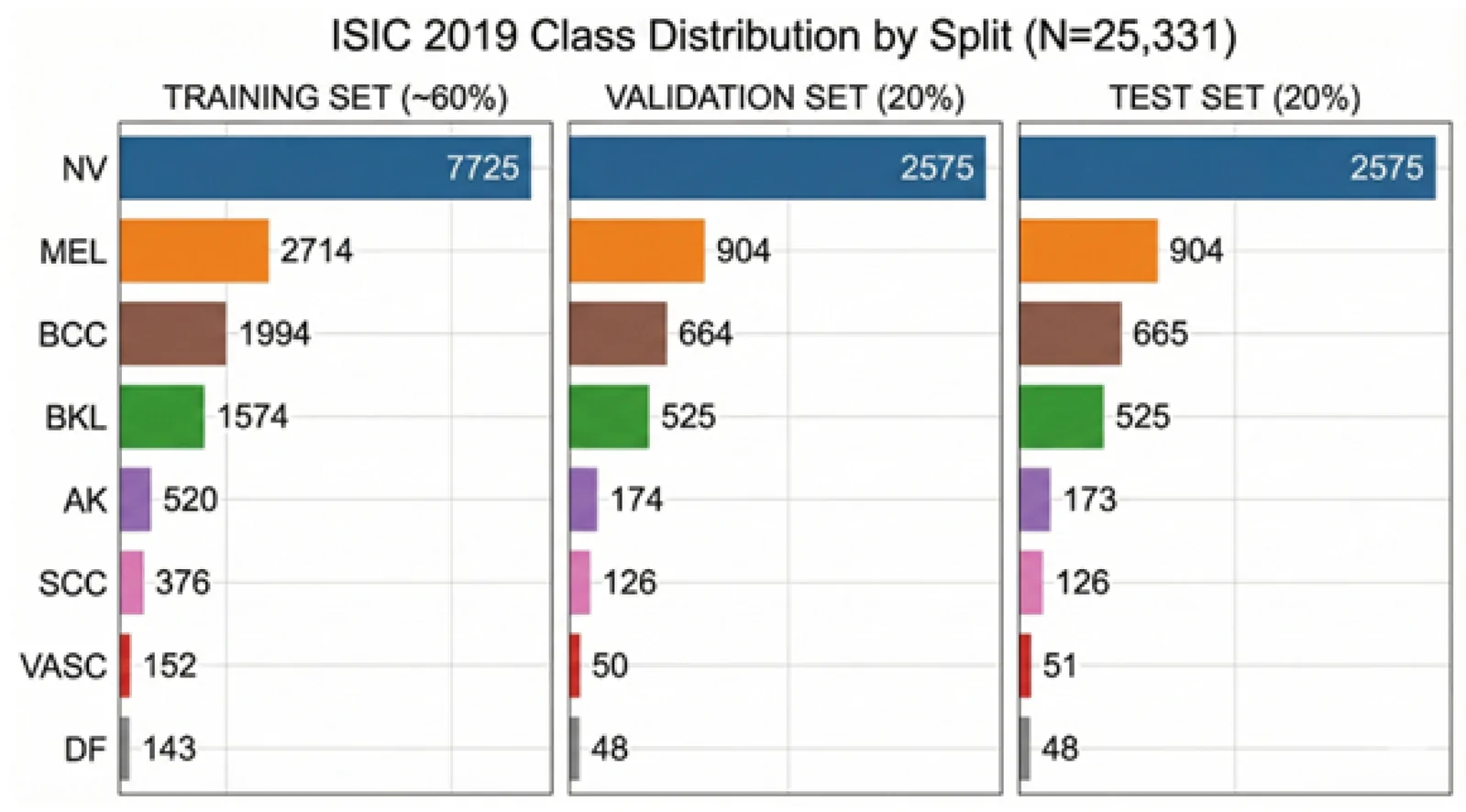
Class distribution across the training, validation, and test sets of the ISIC 2019 dataset.

The dataset was partitioned once using a fixed random seed, with 60% allocated to training, 20% to validation, and 20% to testing. A stratified image-level split was adopted to preserve class proportions across subsets and to align with the image-based evaluation protocol of the ISIC 2019 benchmark. Although lesion-level splitting would prevent images of the same lesion from appearing in different subsets, it was not feasible here because lesion identifiers were unavailable for some images. Image-level splitting was therefore used as a practical choice consistent with the study’s evaluation protocol. Post-selection retraining procedure preserves test-set independence provided that test results are not used to revise the model or its configuration [34]. Generalization was further assessed using the official ISIC 2019 test set and the independent PH^2^ dataset. Figure 2 illustrates the class distribution across the three development subsets.

### 3.2. Evaluation Datasets

#### 3.2.1. ISIC 2019 Official Benchmark Set

To assess generalization of the proposed method on the official ISIC 2019 Benchmark test set, the original test set of 8238 images was used for an independent evaluation. The test set includes an additional unknown (UNK) category, introduced in the challenge to represent out-of-distribution lesions. Since the proposed framework was designed for the eight diagnostic categories available in the training set (MEL, NV, BCC, AK, BKL, DF, VASC, and SCC), UNK samples were excluded. This subset comprised 6,191 images distributed across eight classes: NV (2495), MEL (1327), BCC (975), BKL (660), AK (374), SCC (165), VASC (104), and DF (91).

For the independent benchmark evaluation, the original development partitions were merged into a dataset containing 25,331 images and evaluated using 5-fold Group K-Fold cross-validation. Feature normalization, PCA, probability calibration, and decision-threshold selection were performed exclusively within each training-validation fold to prevent information leakage. Final predictions were obtained by averaging the outputs of the five fold-specific ensembles.

#### 3.2.2. PH^2^ Dermoscopic Image Dataset

To assess the generalization capability of the proposed framework on an independent data source, the PH^2^ dataset was used for external validation [35]. PH^2^ consists of high-quality dermoscopic images with clinical diagnoses and expert annotations that closely align with the diagnostic domain and feature space of the ISIC 2019 dataset. This domain proximity makes PH^2^ a rigorous testbed for verifying the true generalization capacity of our model across unseen clinical environments and hardware scanners.

PH^2^ contains 200 dermoscopic images, including 80 common nevi, 80 atypical nevi, and 40 melanoma cases. Since the proposed framework was primarily developed for eight-class classification on ISIC 2019, a binary evaluation protocol was adopted for PH^2^. For that purpose, the ISIC 2019 MEL class was mapped to the melanoma, while the remaining seven lesion types were grouped into the non-melanoma class. Accordingly, common and atypical nevi in PH^2^ were assigned to the non-melanoma class, resulting in 160 non-melanoma and 40 melanoma lesions. Since the PH^2^ dataset does not provide the patient metadata required by the proposed multimodal framework, the model was adapted for external validation using visual features only. Accordingly, the metadata branch was excluded, and predictions were generated based solely on the visual representations extracted by the image-based backbones.

### 3.3. Initial Model Screening and Backbone Selection

The initial CNN screening used EfficientNetV2M [36] as a baseline because of its favorable balance between predictive performance and model size. In our previous work, EfficientNetV2 was investigated for skin lesion classification, and the positive contribution of data augmentation was demonstrated [37]. This study provided an initial reference for exploring alternative visual architectures.

For Transformer selection, four architectures representing different design paradigms were evaluated: ViT [8], DeiT [38], Twins Transformer [39], and Swin Transformer [12]. All candidates were trained using the same training-validation partitions and a unified hyperparameter configuration. The validation F1-score was used as the primary selection metric due to class imbalance between skin lesions. ViT, DeiT, Twins Transformer, and Swin Transformer achieved F1-scores of 0.81, 0.82, 0.82, and 0.84, respectively. Swin Transformer with the highest performance was selected for subsequent optimization and integration.

The final backbone configuration was extended to include Swin Transformer, EVA-02, and CoAtNet, representing hierarchical shifted-window attention, large-scale Transformer representation learning, and convolution–attention hybrid modeling, respectively. This configuration was intended to combine distinct architectural characteristics within a decision-level ensemble. The final selection thus incorporated architectural heterogeneity alongside predictive performance, with the potential benefit of reducing correlated errors across ensemble members.

### 3.4. Pre-Processing and Augmentation

All pre-processing steps were performed with a fixed random seed (42) to ensure methodological consistency and reproducibility.

Raw dermoscopic images were resized and normalized according to the input resolution required by each backbone model. All images were rescaled with antialiasing enabled and normalized using ImageNet statistics (mean = [0.485, 0.456, 0.406]; std = [0.229, 0.224, 0.225]).

During training, several data augmentation techniques were applied to improve generalization capability, including random rotation (±30°), horizontal/vertical flipping (*p* = 0.5), brightness/contrast/saturation adjustment (±0.2), and hue variation (±0.1). For validation and test samples, only deterministic operations (Resize → ToTensor → Normalize) were used. Data augmentation was employed solely to improve model generalization and robustness, whereas class imbalance was addressed separately using a weighted focal loss during training.

No explicit color standardization or white balance correction was applied; therefore, inter-device variations were intentionally preserved to enhance the model’s robustness and generalizability under real clinical conditions [19].

### 3.5. Model Implementation

#### 3.5.1. Image-Based Feature Extraction

To represent both structural and contextual properties of dermoscopic lesions, three complementary visual backbones were adopted: Swin-B Transformer, EVA-02 Vision Transformer, and CoAtNet. These models respectively emphasize hierarchical local attention, global self-attention, and a convolution–attention hybrid structure, providing diverse yet synergistic perspectives on lesion representation.

All three networks were fine-tuned on the ISIC 2019 dataset through a two-stage progressive-resolution process (i.e., 384→768 for Swin-B, 448→672 for EVA-02, and 224→672 for CoAtNet). This incremental strategy allowed the models to first learn global contextual cues at lower resolutions and later refine fine-grained local patterns at higher ones.

Optimization relied on the AdamW algorithm combined with a cosine-annealed learning-rate schedule. To mitigate the impact of class imbalance, a progressive re-weighting scheme was applied. During the early epochs, standard cross-entropy loss guided learning; in later phases, it was replaced with a class-balanced focal loss (γ≈2,β≈0.999). Validation performance was monitored using the macro-F1 metric, and the model checkpoint achieving the highest score was selected as the final configuration.

Subsequently, pre-logit activations were extracted from each backbone for the training, validation, and test sets under inference mode without gradient computation. These fixed high-dimensional feature representations were stored and later used in the meta-learning and ensemble fusion stages.

#### 3.5.2. Feature Extraction and Meta-Aggregation

Pre-logit features were extracted from the three backbones (Swin-B, EVA-02, and CoAtNet). The extracted feature dimensions were 1024 for Swin-B, 768 for both EVA-02 and CoAtNet. In addition to these visual features, patient-specific metadata comprising age and anatomical region were incorporated into the model. After encoding, age was represented as a 9-dimensional one-hot vector, while anatomical region was encoded using a 10-category vocabulary. Accordingly, the augmented feature vector for each backbone was defined as:

(1) $$x_{b}^{\mathrm{aug}}=\left[\;x_{b}\;∥\;a\;∥\;r\;\right]$$

where $x_{b}\in R^{d_{b}}$ denotes the pre-logit visual embedding extracted from backbone *b*, $a\in R^{d_{\mathrm{age}}}$ represents the one-hot encoded age vector, and $r\in R^{d_{\mathrm{region}}}$ denotes the one-hot encoded anatomical region.

While more complex architectures such as gated or attention-based fusion have been proposed, direct concatenation remains a robust and interpretable late-fusion strategy that preserves modality-specific information and supports diagnostic transparency [40,41,42].

After this integration, the feature dimensionality reached 1043 for Swin-B and 787 for both EVA-02 and CoAtNet. The combined visual and metadata features were subsequently processed through a two-stage statistical pipeline. First, a StandardScaler was fitted exclusively on the training set for each backbone to standardize all features to zero mean and unit variance. As illustrated in Supplementary Figure S1, PCA was fitted on the standardized training features, and the number of components (*k*) was determined to preserve at least 99% of the cumulative explained variance. The fitted StandardScaler and PCA transformations were then applied to the validation data without refitting. After model selection and hyperparameter optimization, both transformations were refitted on the combined training and validation data, while the selected number of components was kept fixed. The resulting transformations were applied to the held-out test data without further optimization or refitting. This procedure yielded 744 components for Swin-B, 391 for EVA-02, and 18 for CoAtNet. This step was used to reduce the dimensionality of the combined visual and metadata features before training the subsequent meta-learners [43].

#### 3.5.3. RobustMLP: Backbone-Aware Classification and Two-Stage Adaptive Calibration

The PCA-compressed visual and metadata features obtained from the two backbones (Swin-B and EVA-02) were processed using a lightweight multilayer perceptron (MLP), designated as RobustMLP. CoAtNet features, in contrast, were classified using class-balanced L2-regularized logistic regression. Rather than denoting a novel architectural topology, this descriptive label specifies a regularized classification head designed to provide stable discrimination while supporting a dedicated probability calibration interface. The module utilizes a minimal single-hidden-layer pipeline:

(2) Linear→BatchNorm→ReLU→Dropout→Linear

This shallow architecture provides a compact nonlinear classifier, with batch normalization and dropout serving as regularization mechanisms.

Training was performed on the training subset using a label-smoothed cross-entropy objective to stabilize optimization and mitigate overconfident predictions. Validation data were used for model-specific hyperparameter selection where applicable and for temperature calibration.

Following classifier optimization, calibration was applied to mitigate the systematic overconfidence inherent in deep neural networks [32,44]. Because heterogeneous visual backbones (Swin-B, EVA-02, and CoAtNet) exhibit distinct structural biases, their raw logit distributions often exhibit systematic confidence differences. To map these outputs onto a comparable confidence scale prior to ensemble fusion, the framework adopts a decoupled Two-Stage Temperature Scaling (T-over-T) protocol:

- Stage 1—Backbone-Specific Temperature Scaling ($T_{i}$):
  For each backbone, a temperature parameter was selected by minimizing the Negative Log-Likelihood (NLL) on the corresponding validation split:
  (3) $$\begin{array}{ccc} T_{i}^{*} & = & \arg\min_{T\in [0.70,1.50]}\mathrm{NLL}\left(y_{\mathrm{val}},\mathrm{softmax}\left(\frac{z_{i}}{T}\right)\right). \end{array}$$
  The selected backbone-specific temperature was then applied to the corresponding validation and external-test predictions prior to ensemble fusion.
- Stage 2—Ensemble Temperature Scaling ($T_{\mathrm{ens}}$):
  After the backbone-specific calibration and NLL-based probability fusion, a second temperature parameter was optimized for the ensemble on the corresponding validation set:
  (4) $$\begin{array}{ccc} T_{\mathrm{ens}}^{*} & = & \arg\min_{T\in [0.70,1.50]}\mathrm{NLL}\left(y_{\mathrm{val}},\mathrm{softmax}\left(\frac{\ln(p_{\mathrm{ens},\mathrm{val}})}{T}\right)\right). \end{array}$$
- Final Calibrated Probability Mapping:
  The resulting ensemble temperature was subsequently applied to the fused ensemble output to obtain the final calibrated class probabilities:
  (5) $$\begin{array}{ccc} p_{cal} & = & \mathrm{softmax}\left(\frac{\log(p_{\mathrm{ens}})}{T_{\mathrm{ens}}^{*}}\right). \end{array}$$

This framework applies a two-stage validation-based temperature scaling strategy [45]. This design helps reduce probability misalignment among heterogeneous ensemble members, allowing varying distributions to be harmonized onto a shared confidence scale while preserving the complementary representation strengths of individual backbones. Empirically, the calibration strategy refined the confidence estimates of individual models and the fused ensemble, with the final calibration applied after probability fusion [46].

#### 3.5.4. Baseline Classifiers, Meta-Ensemble Design, and Reproducibility

The calibrated output probabilities from the three base models (Swin–RobustMLP, EVA–RobustMLP, CoAtNet–LogReg) were then combined into a validation-driven ensemble. The probability of the final ensemble was defined as

(6) $$P_{\mathrm{final}}=w_{\mathrm{Swin}}P_{\mathrm{Swin}}+w_{\mathrm{EVA}}P_{\mathrm{EVA}}+w_{\mathrm{CoAt}}P_{\mathrm{CoAt}},$$

where $w_{\mathrm{Swin}},w_{\mathrm{EVA}},w_{\mathrm{CoAt}}>0$ and $w_{\mathrm{Swin}}+w_{\mathrm{EVA}}+w_{\mathrm{CoAt}}=1$.

A minimum lower bound of 0.05 was imposed on the $w_{\mathrm{CoAt}}$ during the search to prevent its contribution from collapsing to zero. This design choice preserved the unique error profile of the convolutional backbone, particularly in melanocytic and keratinocytic subclasses, within the ensemble. In our experiments, the features produced by the Swin and EVA pipelines tended to dominate the ensemble and could otherwise suppress the influence of the CoAtNet branch. Although the standalone performance of the CoAtNet classifier was less impressive than that of the other two models, its complementary structure improved overall robustness of ensemble. The final weights were selected as the combination that minimized the NLL on the validation set. Class weights were set in a balanced manner to partially compensate for class imbalance, and the regularization strength *C* was optimized over a validation grid search, followed by temperature scaling via NLL minimization.

### 3.6. Experimental Setup

All experiments were conducted using PyTorch 2.11.0 on an NVIDIA A100 GPU, with a fixed random seed and deterministic execution enabled. The architecture-specific hyperparameters and the settings for feature preprocessing, calibration, ensemble optimization, and evaluation are summarized in Supplementary Table S1 and Table S2, respectively.

### 3.7. Code Availability and Reproducibility

The source code for the proposed framework, including feature extraction, model training, ensemble fusion, probability calibration, and evaluation procedures, is available in the project’s GitHub repository: https://github.com/ozanalptekin45/skin-lesion-calibrated-ensemble (accessed on 23 October 2026). The repository includes the experimental notebooks, configuration details, and instructions intended to support reproducibility.

## 4. Results

### 4.1. Individual Transformer Performances

Table 1 summarizes the performance of the three backbone models on the ISIC 2019 test set. EVA-02 achieved the highest macro-F1 score (0.855), while Swin-B obtained the highest accuracy (0.897). CoAtNet showed slightly lower standalone performance but remained competitive, while offering an architecturally distinct hybrid convolutional-attention design. Representative Grad-CAM and EigenCAM visualizations for all three backbones are provided in Supplementary Figures S2–S7, including three correctly classified and three misclassified lesions, illustrating the lesion regions that contribute most strongly to model predictions. Additional analyses reported in Supplementary Table S3 indicate that Swin-B and EVA-02 maintain relatively consistent performance from validation to test data. CoAtNet achieved lower overall performance but remained a potentially complementary backbone. All three backbones achieved strong discriminative performance, with macro-AUC values exceeding 0.96. Class-wise AUC results are reported in Supplementary Table S4. Statistical significance analyses (Supplementary Table S5) indicate no significant performance difference between Swin-B and EVA-02, whereas both models significantly outperform CoAtNet across multiple evaluation criteria.

Overall, these results demonstrate that Swin-B and EVA-02 form a statistically similar and highly reliable backbone pair for dermoscopic lesion representation. Although CoAtNet shows lower standalone performance, its distinct error characteristics support its inclusion as a complementary component within the proposed ensemble. Consequently, individual model evaluations serve as an essential standalone baseline, contextualizing the relative gains achieved by the downstream fusion and calibration strategies.

### 4.2. Comparison of Early and Late Fusion

Before integrating ISIC 2019 metadata, we conducted a set of experiments to better characterize the discriminative limits of representations derived solely from visual data. Table 2 summarizes the performance of individual backbones, early fusion, and optimized late-fusion strategies on the test set. The test-set results were retained to compare the selected late-fusion model against single-model and early-fusion configurations. In the early-fusion setting, feature embeddings extracted from Swin-B, EVA-02, and CoAtNet were concatenated and processed by conventional classifiers. The best early-fusion result was obtained using weighted kNN (k=15) on the combined Swin-B and EVA-02 embeddings, achieving 0.906 accuracy and 0.877 macro-F1.

The fusion strategy was selected based on validation-set experiments, considering individual backbone performance (Supplementary Table S3) and pairwise prediction diversity (Supplementary Table S6). Rather than training a single classifier on the merged feature space, each backbone was paired with its own classification head and calibrated independently before probability aggregation. The selected fusion configuration was then evaluated on the independent test set, achieving an accuracy of 0.911 and a Macro-F1 score of 0.883 (Table 2).

Validation-set performance comparisons and pairwise diversity analyses are reported in Supplementary Tables S3 and Table S6, respectively. The Q-statistics (0.932–0.938) indicate high agreement among the backbones, whereas the disagreement ratios (7.7–8.6%) suggest a limited degree of prediction diversity. Although the backbones exhibit substantial agreement, their disagreements may still provide complementary information. Therefore, ensemble integration was investigated to determine whether combining the backbones could improve predictive performance. Individual backbone results on the independent test set, including class-wise AUC performance, are reported in Supplementary Tables S3 and S4.

### 4.3. Optimization of Feature Representation and Class Balancing

Following the identification of the visual ensemble, the interconnected challenges of high feature dimensionality (>2500) and severe class imbalance in the ISIC 2019 dataset required mitigation prior to the final classification stage.

To prevent overfitting, we evaluated linear dimensionality reduction via Principal Component Analysis (PCA), configured to preserve 99% of the total variance, against non-linear compression using a Deep Embedded Autoencoder (DEA) with a symmetric bottleneck. As summarized in Table 3, PCA-based configurations achieved Macro-F1 scores comparable to those of the DEA configurations, with PCA-0.99 combined with SMOTE yielding the highest score (0.903). SMOTE was evaluated as a potential resampling strategy in the reduced feature space; however, the experiments showed no meaningful performance improvement. Therefore, SMOTE was not included in the final framework, keeping the preprocessing pipeline simpler and computationally lighter. Consequently, PCA was selected as the preferred reduction route.

### 4.4. Ensemble Strategy and Probability Calibration

Table 4 compares the ensemble aggregation strategies evaluated on the validation set. Hard voting (Variant C) produced the weakest performance, achieving a Macro-F1 score of 0.893. In contrast, soft-voting approaches consistently yielded better results, indicating that preserving probability information is beneficial for distinguishing visually similar lesion categories.

Among the evaluated configurations, Variant A (fixed-weight soft voting) achieved the highest validation accuracy (0.927), while Variants A and D achieved the highest Macro-F1 score (0.905). Based on the validation results, Variant A was selected as the preferred ensemble aggregation strategy because it achieved the highest accuracy while maintaining a Macro-F1 score equal to that of Variant D.

The selected soft-voting formulation was subsequently used as the aggregation basis for the final model, which was further refined using validation-driven NLL-optimal fusion weights, two-stage temperature calibration, and clinical metadata integration before final test-set evaluation. Consequently, the final test-set results are not expected to be identical to those obtained for Variant A.

### 4.5. Performance Analysis of the Final Ensemble Model

Table 5 summarizes the performance of the final meta-aware ensemble on the ISIC 2019 test set. The proposed framework achieved an overall accuracy of 0.932 and a macro-F1 score of 0.914, indicating strong and balanced performance across all lesion categories.

**Figure 3:**
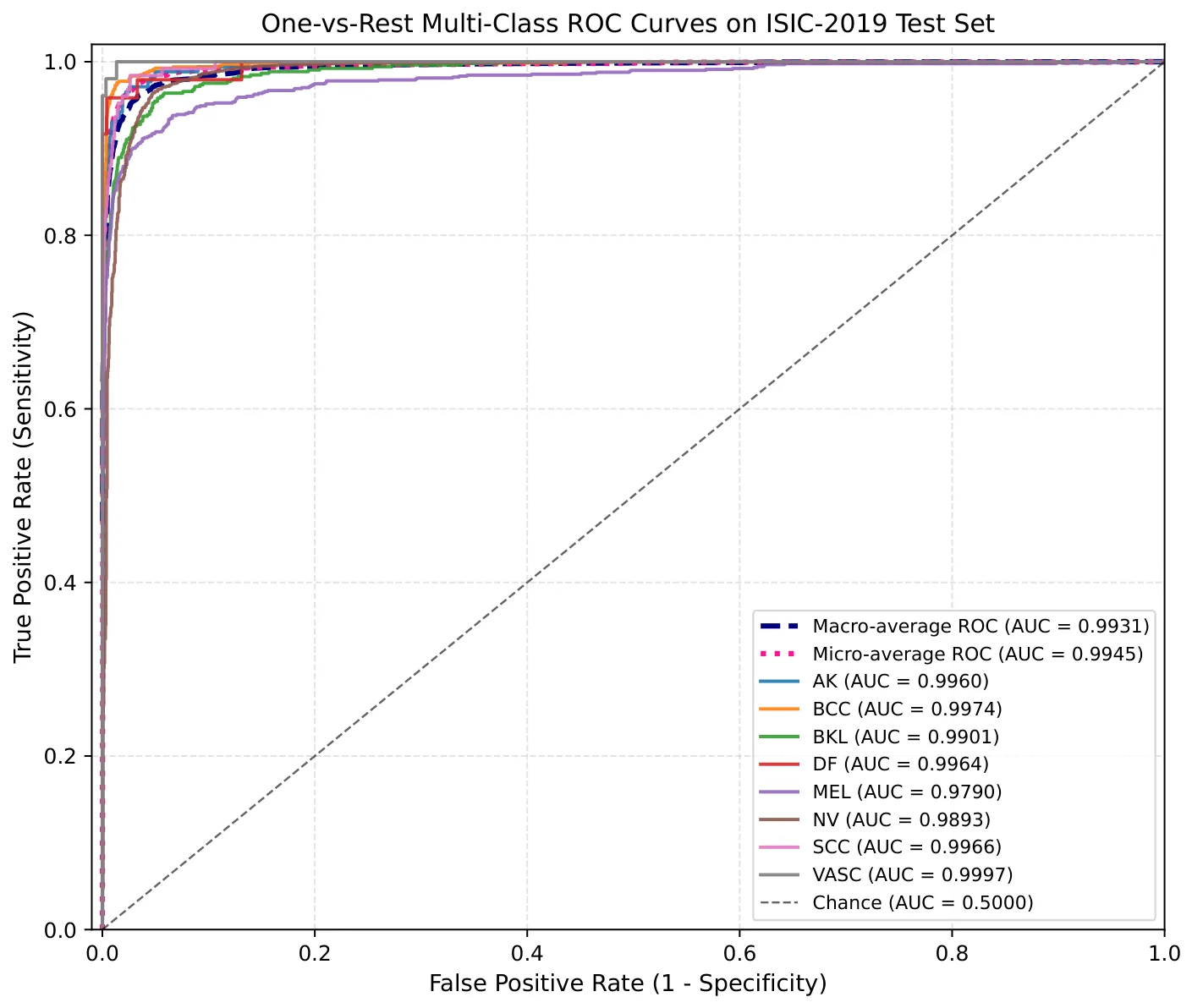
Multi-class one-vs-rest ROC curves of the proposed meta-aware ensemble model on the ISIC 2019 test set.

To better understand how the model behaves across different decision thresholds, we examined the multi-class ROC curves (Figure 3). The macro-AUC was 0.993, and every individual class achieved an AUC above 0.97. Some categories, such as vascular lesions and dermatofibroma, were almost perfectly separable. Even the clinically critical melanoma class achieved an AUC of 0.97.

To gain a clearer understanding of the clinical performance, Table 5 summarizes sensitivity, specificity, and precision for each lesion category. One particularly meaningful outcome is the high specificity achieved for melanoma (0.98). In practice, this means fewer false alarms for benign nevi. Another important result concerns keratinocyte carcinomas. The model achieved an F1-score of 0.867 for actinic keratosis and 0.849 for squamous cell carcinoma. These improvements over vision-only baselines appear to stem from incorporating metadata.

**Figure 4:**
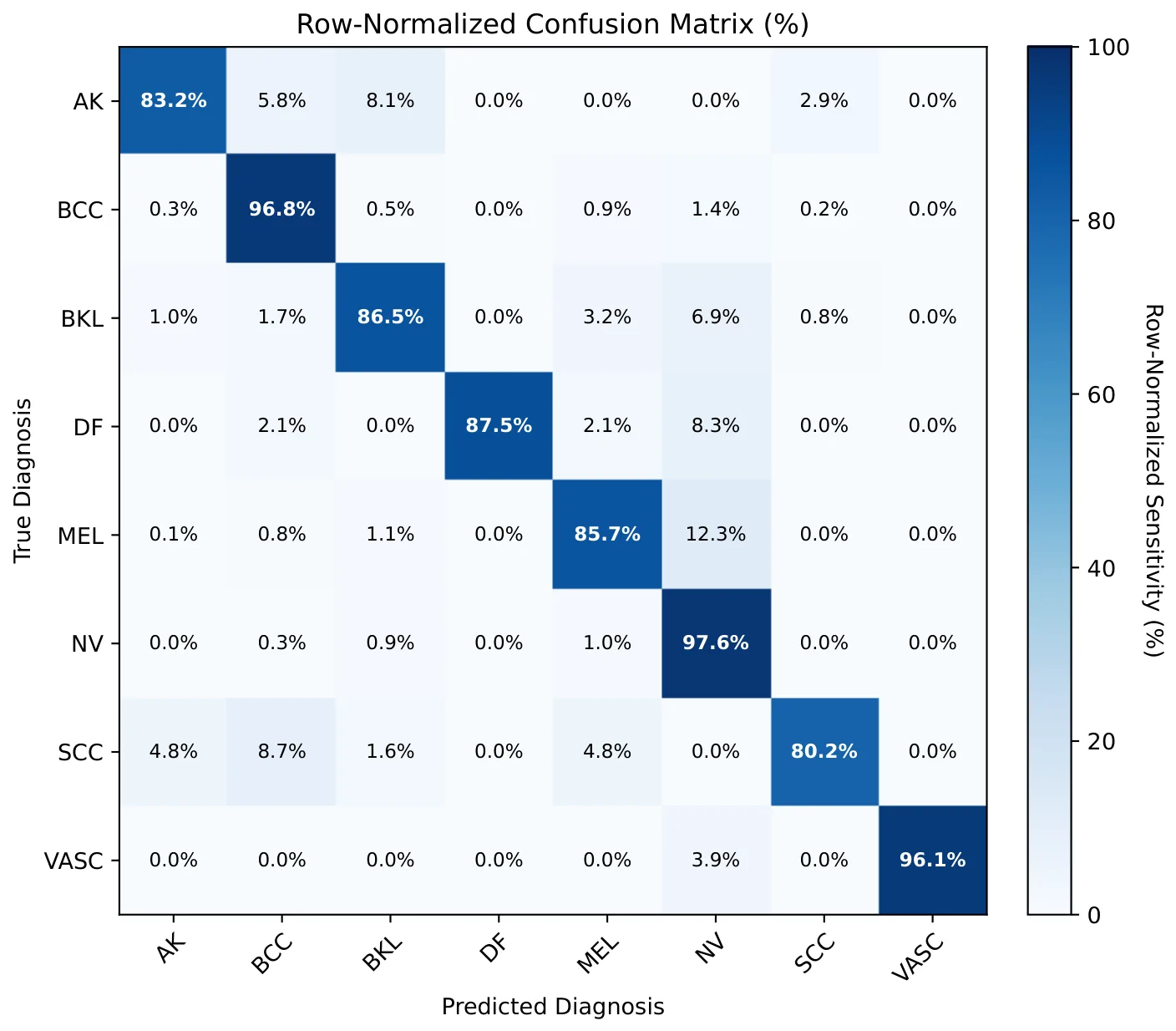
Normalized confusion matrix of the proposed meta-aware framework on the ISIC 2019 test set.

Figure 4 shows how the model misclassified samples between different lesion classes. Most of the errors follow well-known clinical patterns. Melanoma is sometimes confused with melanocytic nevus which aligns with the biological continuum between atypical nevi and early melanomas [16]. On the other hand, confusion between unrelated categories, such as nevus vs. basal cell carcinoma, was extremely rare. The classes VASC and DF showed zero false positives (precision = 1.0), indicating that their visual signatures (e.g., distinct color tones and specific structural cues) are well captured by the models.

The calibration ablation results (Supplementary Tables S7 and S8) revealed complementary effects of the evaluated calibration strategies. Compared with the uncalibrated baseline, the proposed two-stage temperature scaling pipeline reduced ECE from 0.053 to 0.021, indicating an improved calibration. Compared with the single-stage temperature scaling, the proposed pipeline showed promising improvements in accuracy (0.932 vs. 0.928) and Macro-F1 (0.914 vs. 0.909). Conventional temperature scaling yielded a lower ECE (0.015 vs. 0.021). However, the proposed calibration-and-fusion strategy improved classification performance and selected probability-quality metrics, indicating that it provides a favorable balance between accuracy and calibration quality and is a viable alternative to single-stage scaling.

Finally, we evaluated the reliability of the predicted probability estimates using calibration analysis. Calibration analysis (Supplementary Figure S8) demonstrated a balanced agreement between predicted confidence and empirical accuracy, yielding an ECE of 0.021±0.005. Calibration remained particularly strong at higher confidence levels, supporting the reliability of the predicted probabilities for clinical decision support.

### 4.6. External Validation

#### 4.6.1. Official ISIC 2019 Benchmark Set

Table 6 presents the benchmark performance alongside representative literature benchmarks and leaderboard submissions. The BAcc-optimized rule achieved the highest balanced accuracy (0.650) and Macro-F1 (0.657) among the evaluated configurations of the proposed model. Although this balanced accuracy exceeds that of the ISIC 2019 challenge winner (DAISYLab: 0.636), the comparison should be interpreted cautiously, as UNK samples were excluded from evaluation in the present study. Accordingly, the proposed framework performs at least on par with the challenge-winning method, although differences in evaluation protocol preclude a definitive ranking. In addition, compared with the recent hybrid framework reported by Aktan et al. [47], the proposed framework achieved higher balanced accuracy and weighted-F1 while maintaining comparable overall accuracy and macro-AUC. These findings indicate that the proposed framework generalizes well to an independent benchmark and that combining heterogeneous visual representations, probability calibration, and clinical metadata benefits skin lesion classification, particularly for minority classes.

The lesion class-wise ROC-AUC and sensitivity results are reported in Supplementary Tables S9 and S10, respectively. Relative to the reported benchmark results, the proposed model achieved competitive ROC-AUC performance across several lesion categories, including AK, BCC, BKL, and SCC. Complementing this, the BAcc-optimized decision rule achieved sensitivities of 0.736 for melanoma and 0.648 for squamous cell carcinoma, suggesting its potential to support the detection of clinically important malignant and pre-malignant lesions.

#### 4.6.2. PH^2^ Dataset

The same protocol with the ISIC 2019 benchmark evaluation was followed in the PH^2^ dataset. This external dataset remained completely isolated from the model development and other optimization steps. Final predictions were obtained by averaging the outputs of the five fold-specific ensembles.

As summarized in Table 7, the proposed framework achieved an accuracy of 0.955, a macro-F1 score of 0.932, and a ROC-AUC of 0.962 on the PH^2^ dataset. The model correctly identified 37 of 40 melanoma cases (sensitivity = 0.925) while maintaining a specificity of 0.963. In addition, the ensemble demonstrated strong probability calibration, achieving an ECE of 0.027, a Brier score of 0.096 and an NLL of 0.184.

For comparison, Abayomi-Alli et al. [49] reported an accuracy of 0.922, a macro-F1 score of 0.808 and sensitivity of 0.801 on the PH^2^ dataset using covariant SMOTE augmentation with SqueezeNet. AHA-Net [50] achieved the same accuracy, macro-F1 scores of 0.989, and an ROC-AUC of 0.997 on PH^2^. Unlike our study, the models were trained and evaluated on PH^2^ under an in-domain protocol rather than through independent external validation. Golkarieh et al. [51] proposed a semi-supervised GAN for melanoma detection, trained on ISIC-2020 and evaluated on the PH^2^ dataset. They reported an accuracy of 0.895 and an ROC-AUC of 0.866. Unlike approaches evaluated solely in-domain, their study also provides a cross-dataset assessment. Similarly, Chatziioannidis et al. [52] introduced an edge-deployable pipeline combining ResNet-50 and MedFusionNet in a stacking framework. Evaluated on PH^2^ as a held-out external cohort, their ensemble achieved an ROC-AUC of 0.915, an accuracy of 0.810, a sensitivity of 0.850, and a specificity of 0.800 at the frozen decision threshold (θ=0.20).

These results demonstrate that the proposed framework generalizes effectively to an independent external dataset while maintaining strong discrimination and well-calibrated probability estimates beyond the ISIC 2019 domain.

## 5. Discussion

### 5.1. Comparison with Previous Studies

As summarized in Table 8, the proposed meta-aware hybrid framework addresses several gaps that remain unresolved in prior studies. Many single-backbone CNN or transformer-based models, including Swin Transformer [27], EfficientNet B6 [31], and recent hybrid attention-enhanced architectures [28], achieve strong overall accuracy; however, they often exhibit inconsistent sensitivity for visually ambiguous lesion types such as actinic keratosis and squamous cell carcinoma. These approaches rely exclusively on image level features and do not explicitly incorporate clinical metadata. In contrast, the proposed framework systematically integrates metadata through a dedicated fusion strategy, leading to measurable performance gains for these clinically challenging classes.

Although ensemble-based approaches have demonstrated the benefit of combining multiple backbones [20], most existing methods rely on homogeneous or limited diversity architectures. The heterogeneous ensemble adopted in this study integrates Swin-B, EVA-02, and CoAtNet, thereby leveraging complementary inductive biases across architectures. Quantitative analyses further confirm that these backbones exhibit diverse error characteristics, which improves robustness and reduces predictive variance, particularly for keratinizing tumor categories.

Several multi-modal systems incorporate clinical metadata using concatenation or attention mechanisms [23,25], yet they do not explicitly address the reliability of the resulting probabilistic outputs. By contrast, the two-stage adaptive temperature scaling strategy employed in the RobustMLP pipeline is grounded in established calibration theory [32,45] and achieves an expected calibration error of approximately 0.021. This represents a substantial improvement over the implicitly calibrated or uncalibrated predictions reported in earlier studies [21,30].

It is also worth noting that several highly accurate models in the literature evaluate their performance on limited or simplified lesion taxonomies [21,30], which restricts direct comparability with more comprehensive settings. While informative, such simplified settings substantially reduce diagnostic complexity and tend to inflate aggregate performance metrics, limiting direct comparability with full multi-class ISIC 2019 benchmarks. In this study, the evaluation is conducted on the full eight classes of the ISIC 2019 benchmark, where both class imbalance and visual overlap are pronounced [19]. The results demonstrate that strong overall accuracy, balanced performance across minority classes, and clinically meaningful uncertainty estimation can be achieved simultaneously.

A recent study indicated that transformer architectures trained from scratch may struggle under severe class imbalance and limited-data conditions, exhibiting training instability, overfitting, and poor minority-class recognition despite class-weighted optimization [56]. Conversely, studies employing transfer learning and hybrid representation learning have reported more robust performance, including Swin Transformer models initialized with pretrained weights [53], hybrid CNN–ViT architectures that combine local and global feature extraction [54], and fusion-based frameworks integrating complementary CNN and transformer representations [55]. These findings emphasize the importance of architectural diversity, effective representation learning, and clinically relevant contextual information when developing robust skin lesion classification systems. The strong performance observed in the present study suggests that the combination of pretrained visual backbones, metadata integration, probability calibration, and ensemble learning can help mitigate several of these challenges.

### 5.2. Clinical and Methodological Implications

The proposed framework combines three complementary components: heterogeneous visual representations, clinical metadata integration, and calibration-aware prediction. Although Swin-B and EVA-02 achieved the strongest standalone performance, diversity analysis demonstrated that CoAtNet contributed complementary error patterns, particularly for lesion categories characterized by fine-grained textural structures, supporting the value of heterogeneous ensembles beyond individual model performance. This observation is consistent with dermatological studies highlighting the importance of textural cues for keratinizing lesions such as actinic keratosis and squamous cell carcinoma [16,57].

The incorporation of patient age and anatomical region further improved class-balanced performance, particularly for diagnostically challenging lesion categories. These improvements are consistent with established epidemiological knowledge, where lesion prevalence and risk profiles vary substantially across age groups and anatomical locations [58,59]. The results therefore suggest that clinically relevant contextual information provides complementary value beyond visual appearance alone.

In addition to discriminative performance, the framework explicitly addresses probability reliability through the proposed two-stage adaptive temperature scaling strategy. The resulting calibration performance (ECE ≈0.021) indicates good agreement between predicted confidence and observed outcomes, an important requirement for clinical decision-support systems where prediction confidence may directly influence downstream diagnostic decisions [32,60].

On the internal ISIC 2019 hold-out test set, the proposed metadata-aware framework achieved an accuracy of 0.93, a macro-F1 score of 0.91, and a macro-AUC of 0.99. Importantly, the evaluation also included the official ISIC 2019 benchmark set. On this benchmark, the metadata-enhanced ensemble achieved a balanced accuracy of 0.65, which is numerically higher than the reported score of the challenge winner under the primary ranking metric. However, differences in evaluation protocols, including the exclusion of UNK samples in the present study, warrant a cautious interpretation of this comparison. Furthermore, external validation on the independent PH^2^ dataset yielded an accuracy of 0.95 and an ROC-AUC of 0.96.

Beyond its methodological contributions, the proposed framework shows promising potential for clinical use, with encouraging generalization across the ISIC 2019 and PH^2^ datasets, which differ in acquisition conditions. In practice, it could be integrated into teledermatology platforms and primary-care decision-support systems as an automated triage aid, helping prioritize referrals of suspected malignant lesions to dermatologists, reduce diagnostic workload, and support more consistent assessments across settings. These benefits remain contingent on prospective, multi-center validation, evaluation across diverse skin tones, and clinician-in-the-loop oversight, as discussed in the limitations in the next section.

### 5.3. Limitations

Despite its strong performance, the proposed framework has some limitations:

- The relatively lower sensitivity observed for AK and SCC reflects an inherent clinical challenge. These lesions share substantial dermoscopic overlap with other categories, and even experienced dermatologists frequently disagree on their visual assessment [61].
- Although external validation was performed on both the official ISIC 2019 benchmark and the independent PH^2^ dataset, broader multi-center validation remains necessary. Differences in imaging devices, acquisition protocols, patient populations, and geographic distributions may influence model performance in real-world clinical settings.
- The PH^2^ evaluation was conducted in a binary melanoma–non-melanoma setting due to lack of compatible annotations with the ISIC 2019 taxonomy.
- ISIC 2019 contains mainly images of lighter skin tones, and PH^2^ has also a limited diversity. Performance on patients with darker skin tones has not been verified and should be assessed on more diverse cohorts.
- The framework was evaluated retrospectively on curated public datasets. Prospective validation on consecutive patients, with histopathology as the reference standard, is needed to establish a clinical utility.
- The use of three visual backbones increases computational cost compared with single-model approaches. This trade-off may be acceptable in settings where diagnostic reliability is a priority.

## 6. Conclusions

The proposed approach achieved competitive predictive performance and improved selected probability calibration metrics. The integration of metadata proved especially valuable for visually overlapping classes, while ensemble diversity contributed to improved robustness in challenging keratinizing lesions. Furthermore, explainability analyses confirmed that the model focuses on clinically meaningful regions, supporting its potential relevance for real-world decision support.

As a future work, we plan to assess the model across multiple external datasets to better evaluate its performance under varying imaging conditions and population characteristics. Overall, the study provides a reproducible and clinically aligned foundation for future advances in automated dermatological diagnosis.

## Figures and Tables

**Table 1:** Overall performance comparison of single transformer backbones on the test set.

Model	Accuracy	Macro-F1	MCC	Macro-AUC
Swin-B	0.897	0.845	0.840	0.984
EVA-02	0.890	0.855	0.840	0.985
CoAtNet	0.881	0.838	0.820	0.968

Swin-B: base variant of the Swin (shifted window) Transformer; EVA-02: a next-generation Transformer-based visual representation model; CoAtNet: convolution and attention network; Macro-F1: macro-averaged F1-score; MCC: Matthews correlation coefficient; Macro-AUC: macro-averaged area under the receiver operating characteristic curve.

**Table 2:** Performance analysis of visual feature fusion strategies on the test set.

Strategy	Configuration	Accuracy	Macro-F1	MCC
Early Fusion (Concat)	Swin + EVA	0.906	0.877	0.861
	Swin + EVA + CoAt	0.905	0.876	0.860
Late Fusion (Ensemble)	Swin + EVA + CoAt	0.911	0.883	0.868

Swin: base variant of the Swin (shifted window) Transformer; EVA: a next-generation Transformer-based visual representation model; CoAt: convolution and attention network; Macro-F1: macro-averaged F1-score; MCC: Matthews correlation coefficient.

**Table 3:** Performance comparison of different feature reduction strategies on the validation set.

Strategy	Configuration	SMOTE	Accuracy	Macro-F1
Linear Projection	PCA (variance = 0.99)	Yes	0.926	0.903
Linear Projection	PCA (variance = 0.99)	No	0.926	0.902
Non-linear	DEA (dimension = 1024)	No	0.927	0.902
Non-linear	DEA (dimension = 2048)	Yes	0.925	0.902
Non-linear	DEA (dimension = 512)	Yes	0.924	0.897

PCA: principal component analysis; DEA: deep embedded autoencoder.

**Table 4:** Comparative analysis of ensemble aggregation strategies on the validation set.

Variant	Strategy	Voting	Accuracy	Macro-F1
A	Fixed Weights	Soft	0.927	0.905
B	Dynamic Weights	Soft	0.926	0.903
C	Majority Rule	Hard	0.921	0.893
D	Expanded Ensemble	Weighted	0.924	0.905

**Table 5:** Class-wise and overall diagnostic performance of the proposed meta-aware framework on the ISIC 2019 test set after integration of clinical metadata (age and anatomical region).

Diagnosis Class	Sensitivity	Specificity	Precision	F1-Score
Actinic Keratosis (AK)	0.832	0.997	0.906	0.867
Basal Cell Carcinoma(BCC)	0.968	0.989	0.932	0.950
Benign Keratosis (BKL)	0.865	0.988	0.895	0.880
Dermatofibroma (DF)	0.875	1.000	1.000	0.933
Melanoma (MEL)	0.857	0.986	0.932	0.893
Melanocytic Nevus(NV)	0.976	0.935	0.940	0.957
Squamous Cell Carcinoma (SCC)	0.802	0.998	0.902	0.849
Vascular Lesion (VASC)	0.961	1.000	1.000	0.980
Overall performance with 95% Stratified Bootstrap CI				
Overall Accuracy	0.932 ± 0.007			
Macro F1-score	0.914 ± 0.013			
Balanced Accuracy	0.892 ± 0.018			
Macro ROC AUC	0.993 ± 0.001			

**Table 6:** Comparison with representative studies and official leaderboard submissions evaluated on the ISIC 2019 benchmark set [48]. The results of the proposed model are reported with 95% stratified bootstrap confidence intervals.

Method	Inference Strategy	N	Accuracy	Balanced Acc.	Weighted F1	Macro AUC
DAISYLab (#1) [20]	CNN Ens. + Metadata	8238	–	0.636	–	0.913
DysionAI (#2) [48]	Deep CNN Ensemble	8238	–	0.607	–	0.780
AImageLab (#3) [48]	CNN Ensemble	8238	–	0.593	–	0.886
Aktan et al. [47]	CNN + ViT + MambaVision	6191	0.748	0.607	0.743	0.942
Proposed Model	Standard Argmax	6191	0.756 ± 0.010	0.568 ± 0.021	0.742 ± 0.011	0.934 ± 0.007
Proposed Model	BAcc-Optimized Rule	6191	0.749 ± 0.011	0.650 ± 0.020	0.751 ± 0.011	0.934 ± 0.007

**Table 7:** Performance comparison on the PH^2^ dataset under external and in-domain evaluation protocols. The results of the proposed model (with 95% stratified bootstrap confidence intervals) are provided as external domain validation whereas some previous studies reported their in-domain (only PH^2^) evaluations.

Model	Training/Evaluation	Accuracy	Macro F1	ROC-AUC	Sensitivity
Abayomi-Alli et al. [49]	PH^2^	0.922	0.808	–	0.801
AHA-Net [50]	PH^2^	0.989	0.989	0.997	0.988
Golkarieh et al. [51]	ISIC 2020/PH^2^	0.895	-	0.866	0.910
Chatziioannidis et al. [52]	ISIC 2019 + 2020/PH^2^	0.810	-	0.915	0.850
Proposed Model	ISIC 2019/PH^2^	0.955 ± 0.030	0.932 ± 0.042	0.962 ± 0.041	0.925 ± 0.075

**Table 8:** Comparison with representative studies on skin lesion classification using ISIC-based datasets.

Study	Method/Architecture	Metadata	Accuracy	BACC/Macro-F1	AUC
Gessert et al. [20]	Ensemble (EfficientNet, SENet, ResNeXt)	Yes	–	0.634	0.910
Kassem et al. [21]	Modified GoogleNet (Transfer Learning)	No	0.949	–	–
Yin et al. [23]	DenseNet-169 + MD-Net (MetaNet, MetaBlock)	Yes	0.841	0.856	0.980
Ayas [27]	Swin Transformer (Large-22K)	No	0.972	0.823	–
Saeed et al. [30]	Hybrid (VGG19 + SVM) + Generative AI	No	0.930	–	0.960
Cino et al. [31]	EfficientNet-B6 + TTA + XAI	No	0.976	0.860	0.979
Ozdemir et al. [28]	Hybrid ConvNeXtV2 + Attention	No	0.935	0.918	–
Younas et al. [29]	AIR-CNN (Inception-Residual + Attention)	No	0.916	–	–
Panneerselvam et al. [25]	EfficientNet-B3 + TabNet (Multimodal)	Yes	0.987	0.805	–
Lian et al. [53]	Swin-T + Transfer Learning	No	0.936	0.881	–
Aruk et al. [54]	Hybrid CNN–ViT	No	0.925	0.904	–
AbuAlkebash et al. [55]	CNN–ViT Fusion	No	0.906	0.840	0.980
This study	Meta-aware Hybrid	Yes	0.932	0.914	0.993

BACC: balanced accuracy; Macro-F1: macro-averaged F1-score; AUC: area under the receiver operating characteristic curve.

## Data Availability

The ISIC 2019 training dataset aggregates images from multiple sources. In accordance with the CC BY-NC license attribution requirements, the following attributions are provided (https://challenge.isic-archive.com/data/#2019 (accessed on 1 June 2024)): BCN_20000 Dataset: Copyright Department of Dermatology, Hospital Clínic de Barcelona; HAM10000 Dataset: Copyright ViDIR Group, Department of Dermatology, Medical University of Vienna (https://doi.org/10.1038/sdata.2018.161); MSK Dataset: Copyright Anonymous (https://arxiv.org/abs/1710.05006 (accessed on 23 October 2026); https://arxiv.org/abs/1902.03368 (accessed on 23 October 2026)). The PH^2^ dataset is available from its official source at https://www.fc.up.pt/addi/ph2%20database.html (accessed on 5 May 2025), subject to its applicable terms of use. The code and reproducibility materials of the current study are publicly available at GitHub: https://github.com/ozanalptekin45/skin-lesion-calibrated-ensemble (accessed on 23 October 2026).

## Supplementary Materials

The following supporting information can be downloaded at https://www.mdpi.com/article/10.3390/diagnostics16213613/s1, Figure S1: Feature standardization and PCA workflow, including training-based component selection and final PCA refitting; Figure S2: Grad-CAM and EigenCAM visualizations for the Swin-B model; Figure S3: Grad-CAM visualizations for the EVA-02 model; Figure S4: Grad-CAM visualizations for the CoAtNet model; Figure S5: Grad-CAM and EigenCAM visualizations of a misclassified lesion by the Swin-B model; Figure S6: Grad-CAM and EigenCAM visualizations of a misclassified lesion by the EVA-02 model; Figure S7: Grad-CAM and EigenCAM visualizations of a misclassified lesion by the CoAtNet model; Figure S8: Calibration reliability diagram of the proposed meta-aware ensemble; Table S1: Architecture-specific hyperparameters of the proposed ensemble members; Table S2: Training, feature preprocessing, calibration, ensemble optimization, and evaluation parameters; Table S3: Validation and test performance of the individual backbone models; Table S4: Skin lesion-wise AUC performance of the individual backbone models; Table S5: Pairwise statistical comparison of the visual backbones on the test set; Table S6: Pairwise diversity analysis of the visual backbones on the validation set; Table S7: Calibration ablation results on the independent test set; Table S8: Paired statistical comparisons of the proposed two-stage temperature scaling strategy; Table S9: Class-wise ROC-AUC comparison on the ISIC 2019 benchmark test dataset; Table S10: Class-wise sensitivity comparison on the ISIC 2019 benchmark test dataset.

## Abbreviations

The following abbreviations are used in this manuscript:

AI	Artificial Intelligence
AUC	Area Under the Receiver Operating Characteristic Curve
BACC	Balanced Accuracy
BCC	Basal Cell Carcinoma
CNN	Convolutional Neural Network
ECE	Expected Calibration Error
ISIC	International Skin Imaging Collaboration
MCC	Matthews Correlation Coefficient
MLP	Multilayer Perceptron
NLL	Negative Log-Likelihood
NV	Melanocytic Nevus
PCA	Principal Component Analysis
PH^2^	PH^2^ Dermoscopic Image Dataset
ROC	Receiver Operating Characteristic
SCC	Squamous Cell Carcinoma
SMOTE	Synthetic Minority Over-sampling Technique
SVM	Support Vector Machine
ViT	Vision Transformer
