# Supplementary material for: Calibration-Aware Heterogeneous Multimodal Ensemble for Multi-Class Skin Lesion Diagnosis
Source: Diagnostics (Basel). 2026 Nov 1;16(21):3613. doi: 10.3390/diagnostics16213613 (PMC13762094; doi:10.3390/diagnostics16213613)
Supplement: Supplementary file 1 [file diagnostics-16-03613-s001.zip › Supplementary.pdf]

Supplementary Materials: Supplementary Material

1. Supplementary Tables

Table S1. Architecture-specific hyperparameters of the proposed ensemble members.

Model	Hidden	Dropout	Learning Rate	Weight Decay	Optimizer	Loss	Epochs	Batch Size
Swin-B + RobustMLP	2048	0.1606	3.4141×10^{-4}	1.4883×10^{-4}	AdamW	CE + LS	70	256
EVA-02 + RobustMLP	2048	0.3203	9.7655×10^{-4}	8.2125×10^{-5}	AdamW	CE + LS	70	256
CoAtNet + Log. Reg.	–	–	–	–	L-BFGS	–	–	–

CE: cross-entropy loss; LS: label smoothing (0.05). For CoAtNet, the regularization parameter C was selected from $\{1.6, 2.3402, 3.2\}$ according to validation NLL.

Table S2. Training, feature preprocessing, calibration, ensemble optimization, and evaluation parameters.

Parameter	Setting
Random seed	42
Feature standardization	StandardScaler
PCA	Enabled
PCA variance threshold	$\geq$ 99%
Stage-1 PCA fitting	Training set only
Stage-2 PCA fitting	Training + validation sets
Stage-2 PCA dimensionality	Stage-1 selected k (frozen)
MLP batch size	256
MLP training epochs	70
Label smoothing	0.05
Stage-1 temperature range	0.70–1.50
Stage-1 temperature step	0.02
Stage-2 ensemble temperature range	0.70–1.50
Stage-2 temperature step	0.005
Temperature selection criterion	Validation NLL
Ensemble weight selection criterion	Validation NLL
Ensemble weight grid step	0.02
Minimum CoAtNet ensemble weight	0.05
Final decision rule	arg max of calibrated probabilities
Calibration bins	15
Bootstrap replicates	1,000
Confidence interval	95% stratified percentile bootstrap
Computational platform	Google Colab Pro
GPU	NVIDIA A100

Table S3. Validation and test performance of Swin-B, EVA-02, and CoAtNet on the ISIC2019 dataset. Values are reported as mean ± 95% confidence interval (CI).

Dataset	Model	Accuracy	Macro-F1
VAL	Swin-B	0.907 ± 0.008	0.863 ± 0.017
	EVA-02	0.905 ± 0.008	0.873 ± 0.016
	CoAtNet	0.885 ± 0.009	0.857 ± 0.016
TEST	Swin-B	0.897 ± 0.008	0.845 ± 0.019
	EVA-02	0.890 ± 0.008	0.855 ± 0.017
	CoAtNet	0.881 ± 0.009	0.838 ± 0.019

Table S4. Skin lesion-wise AUC performance of individual backbone models on the test set.

**Model**	**AK**	**BCC**	**BKL**	**DF**	**MEL**	**NV**	**SCC**	**VASC**
Swin-B	0.986	0.995	0.972	0.992	0.966	0.983	0.991	0.992
EVA-02	0.988	0.994	0.972	0.991	0.966	0.979	0.990	0.999
CoAtNet	0.946	0.981	0.955	0.973	0.951	0.973	0.963	0.997

Table S5. Pairwise statistical comparison of the visual backbones on the test set. DeLong's test was used for Macro-AUC, McNemar's test for paired error diversity, and permutation tests for accuracy.

Statistical Test	Comparison Pair	Statistic	p-value
DeLong	Swin-B vs. EVA-02	$Z \approx -0.86$	0.387
	Swin-B vs. CoAtNet	$Z \approx 7.30$	< 0.001
	EVA-02 vs. CoAtNet	$Z \approx 8.12$	< 0.001
McNemar	Swin-B vs. EVA-02	$\chi^2 \approx 3.1$	0.079
	Swin-B vs. CoAtNet	$\chi^2 > 15$	< 0.001
	EVA-02 vs. CoAtNet	$\chi^2 \approx 4.0$	0.049
Permutation	Swin-B vs. EVA-02	ΔAcc $\approx$ 0.7%	0.082
	Swin-B vs. CoAtNet	ΔAcc $\approx$ 1.6%	< 0.001
	EVA-02 vs. CoAtNet	ΔAcc $\approx$ 0.9%	0.049

Table S6. Pairwise diversity analysis of the visual backbones on the validation set.

**Model Pair**	**Q-Statistic**	**Disagreement**
Swin vs. EVA-02	0.935	0.077
Swin vs. CoAtNet	0.938	0.083
EVA-02 vs. CoAtNet	0.932	0.086

Table S7. Calibration ablation results on the independent test set for the multimodal pipeline. ECE was computed using 15 equal-width confidence bins over $[0, 1]$.

**Calibration Pipeline**	**Selected Temperature(s)**	**ECE**	**NLL**	**Brier**	**Acc.**	**BAcc.**	**Macro-F1**
Uncalibrated	–	0.053	0.265	0.114	0.928	0.886	0.906
Conventional TS (Single-Stage)	$T_{\mathrm{conv}} = 0.83$	0.015	0.240	0.110	0.928	0.889	0.909
Two-Stage Scalar TS (Proposed)	$T_{\mathrm{Swin}} = 0.84$ $T_{\mathrm{EVA}} = 0.96$ $T_{\mathrm{CoAtNet}} = 1.50$ $T_{\mathrm{ens}} = 0.895$	0.021	0.239	0.109	0.932	0.892	0.914

Table S8. Paired statistical comparisons of the proposed two-stage temperature scaling strategy.

Comparison	Metric	Δ	95% Bootstrap CI	McNemar p
Two-stage TS vs. uncalibrated				
	Accuracy	$+0.0038$	$[+0.0006, +0.0069]$	0.0248
	Macro-F1	$+0.0080$	$[-0.0009, +0.0117]$	–
	ECE	-0.0316	$[-0.0352, -0.0278]$	–
	NLL	-0.0251	$[-0.0282, -0.0221]$	–
	Brier score	-0.0054	$[-0.0068, -0.0039]$	–
Two-stage TS vs. conventional TS				
	Accuracy	$+0.0038$	$[+0.0006, +0.0069]$	0.0248
	Macro-F1	$+0.0050$	$[-0.0009, +0.0117]$	–
	Brier score	-0.0015	$[-0.0028, -0.0003]$	–
	NLL	-0.0009	$[-0.0030, +0.0012]$	–
	ECE	$+0.0050$	$[+0.0001, +0.0090]$	–

Table S9. The class-wise ROC-AUC comparison on the standard eight target classes of the ISIC 2019 benchmark test dataset.

**Method**	**AK**	**BCC**	**BKL**	**DF**	**MEL**	**NV**	**SCC**	**VASC**
DAISYLab (#1)	0.914	0.949	0.904	**0.979**	0.928	**0.960**	0.938	**0.956**
DysionAI (#2)	0.765	0.868	0.762	0.832	0.808	0.878	0.744	0.797
AImageLab(#3)	0.896	0.947	0.907	0.977	**0.933**	0.954	0.9220	0.938
**Proposed Model**	**0.921**	**0.970**	**0.912**	0.955	0.924	0.956	**0.951**	0.887

Table S10. The class-wise Sensitivity comparison on the standard eight target classes of the ISIC 2019 benchmark test dataset.

**Method**	**AK**	**BCC**	**BKL**	**DF**	**MEL**	**NV**	**SCC**	**VASC**
DAISYLab (#1)	0.484	0.721	0.394	0.578	0.594	0.710	0.439	**0.644**
DysionAI (#2)	**0.596**	**0.841**	**0.562**	0.678	0.675	0.813	0.516	0.604
AImageLab (#3)	0.321	0.853	0.551	0.567	0.684	**0.866**	0.446	0.564
**Proposed Model**	0.551	0.835	0.506	**0.681**	**0.736**	0.840	**0.648**	0.404

2. Supplementary Figures

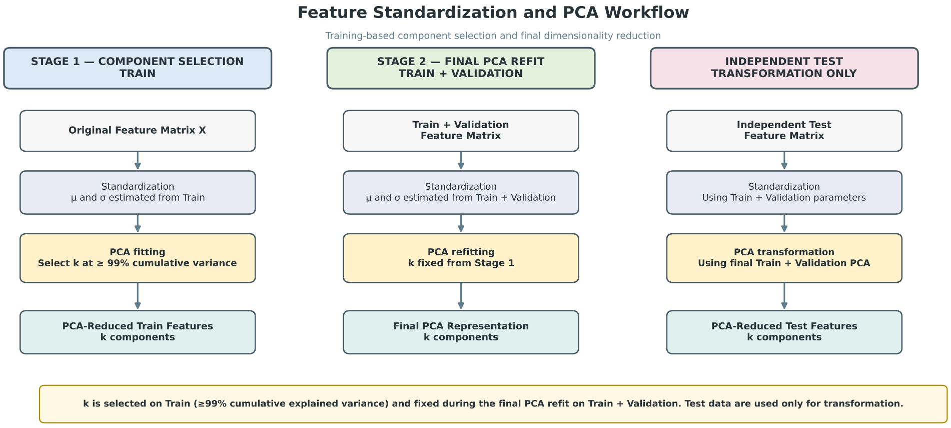

Figure S1. Feature standardization and PCA workflow showing training-based component selection, final PCA refitting with the selected number of components, and transformation of the independent test set.

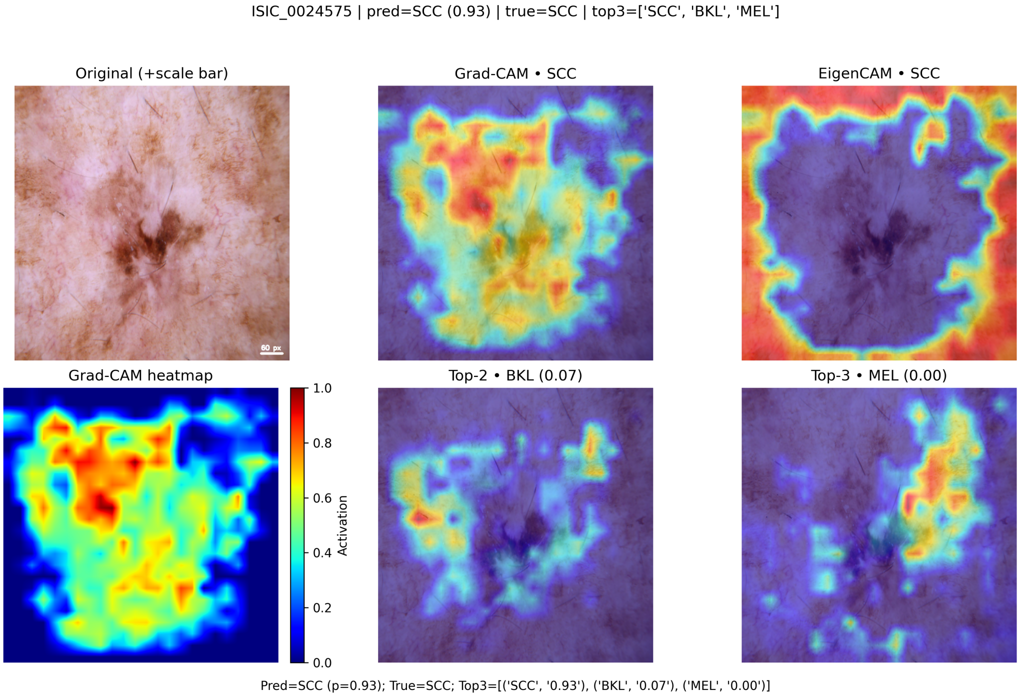

Figure S2. Grad-CAM and EigenCAM visualizations of an SCC lesion using the Swin-B model. This figure shows examples of Grad-CAM heatmaps generated by the Swin-B model. It correctly predicts the lesion, with activation concentrated in areas of irregular pigmentation near its center, while the top-2 and top-3 activation maps show secondary activation in distinct regions for the BKL and MEL classes. These visualizations suggest that the model may capture clinically relevant structural patterns and provide insights into its decision-making process.

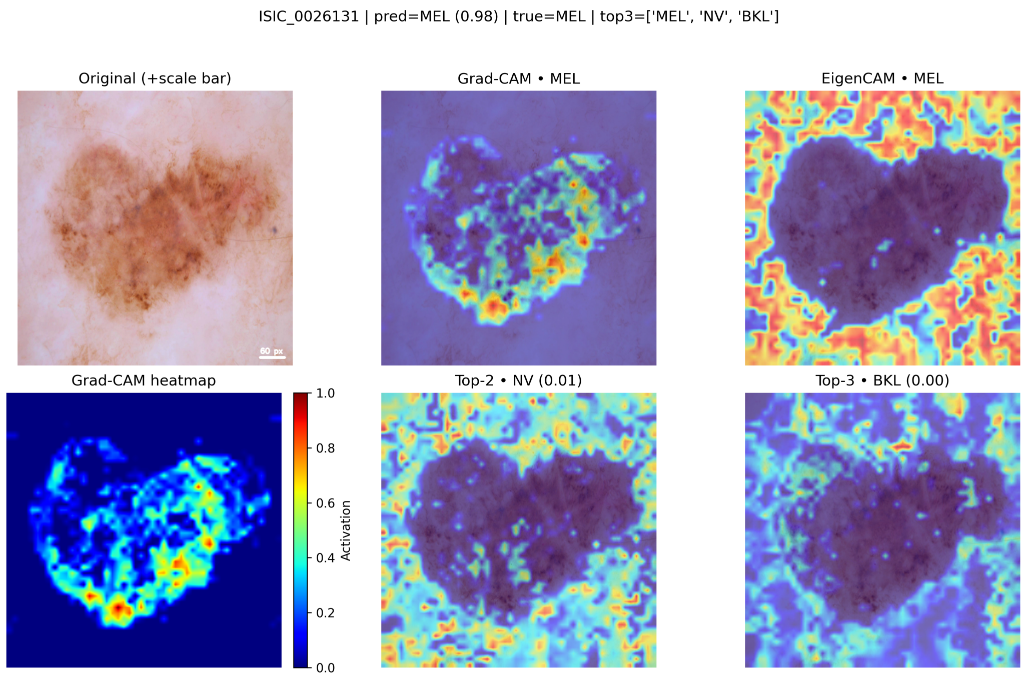

Figure S3. Grad-CAM visualizations for correctly classified lesions obtained using the EVA-02 model. In this figure, EVA-02 predicts melanoma with a confidence score of 98%. As seen in the lower-left panel, the model's activation is concentrated around the asymmetric borders of the lesion and the irregular pigmentation (color) distribution within it. These visualizations appear to be consistent with certain aspects of the ABCDE rule (Asymmetry, Border, Color) used in clinical practice.

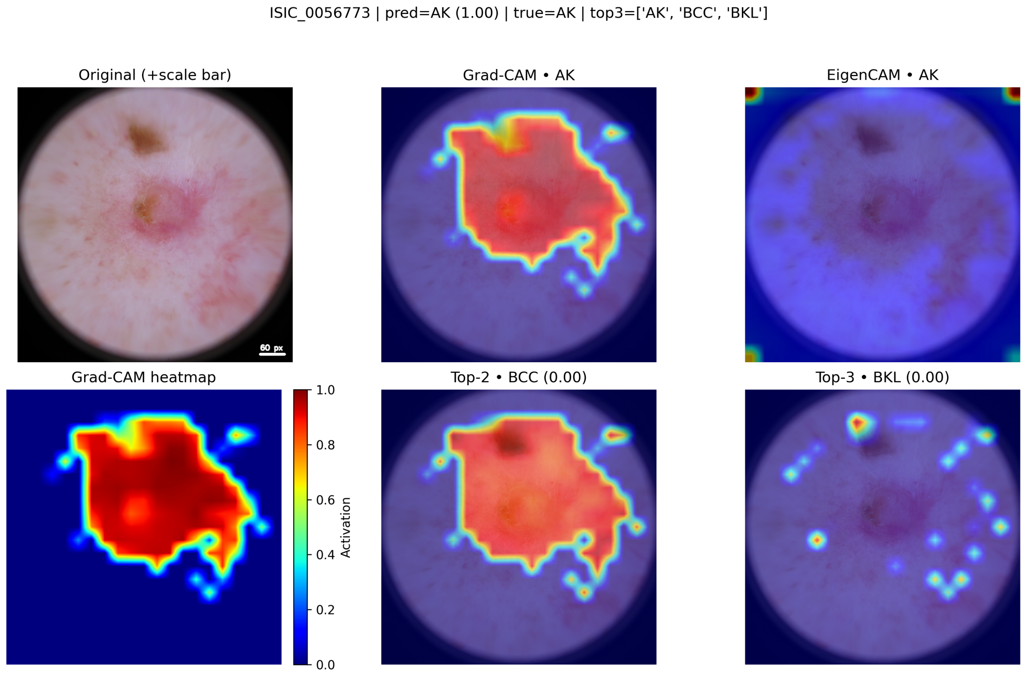

Figure S4. Grad-CAM explainability for a correctly classified Actinic Keratosis (AK) sample using the CoAtNet backbone. Clinically, actinic keratosis is characterized by a superficial, erythematous, and scaly presentation. As illustrated in this figure, the convolutional layers of CoAtNet may encode this fine-grained textural information. The corresponding heatmap reveals that the model's activation is distributed across much of the lesion surface rather than being restricted to isolated focal points. This pattern suggests that the model may use spatially distributed features when classifying the lesion.

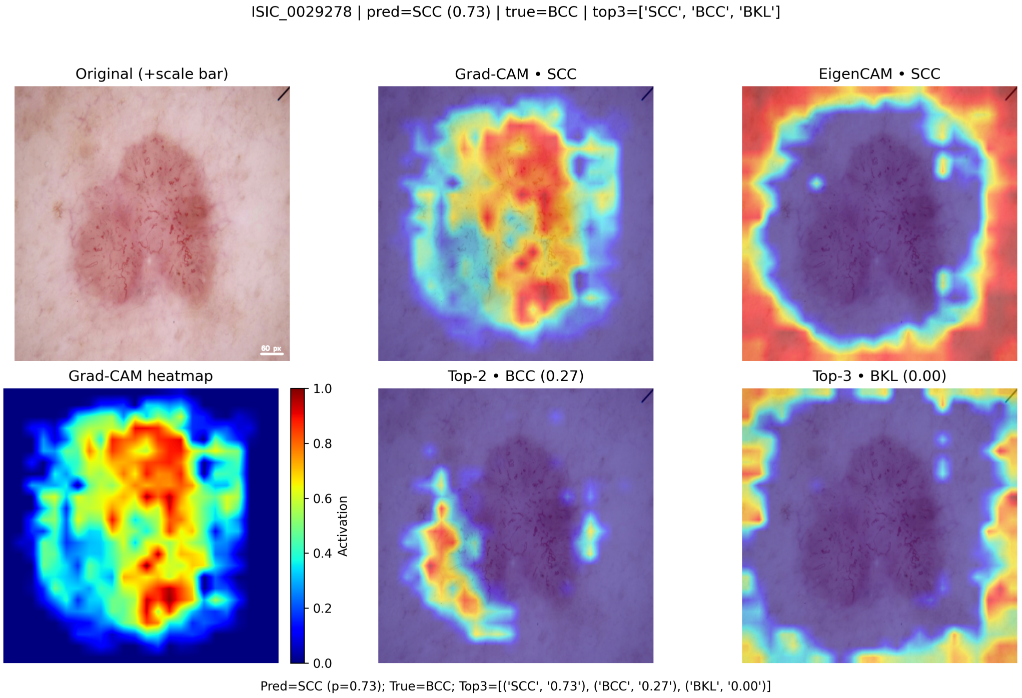

Figure S5. Grad-CAM and EigenCAM visualizations for a misclassified lesion by the Swin baseline model. Grad-CAM highlights central erythematous regions and vascular structures that may be associated with the SCC prediction. EigenCAM shows additional activation near the lesion periphery, suggesting that boundary-related features may also be represented in the model response. These visualizations provide qualitative insights into the misclassification, although they do not establish the specific features responsible for the prediction.

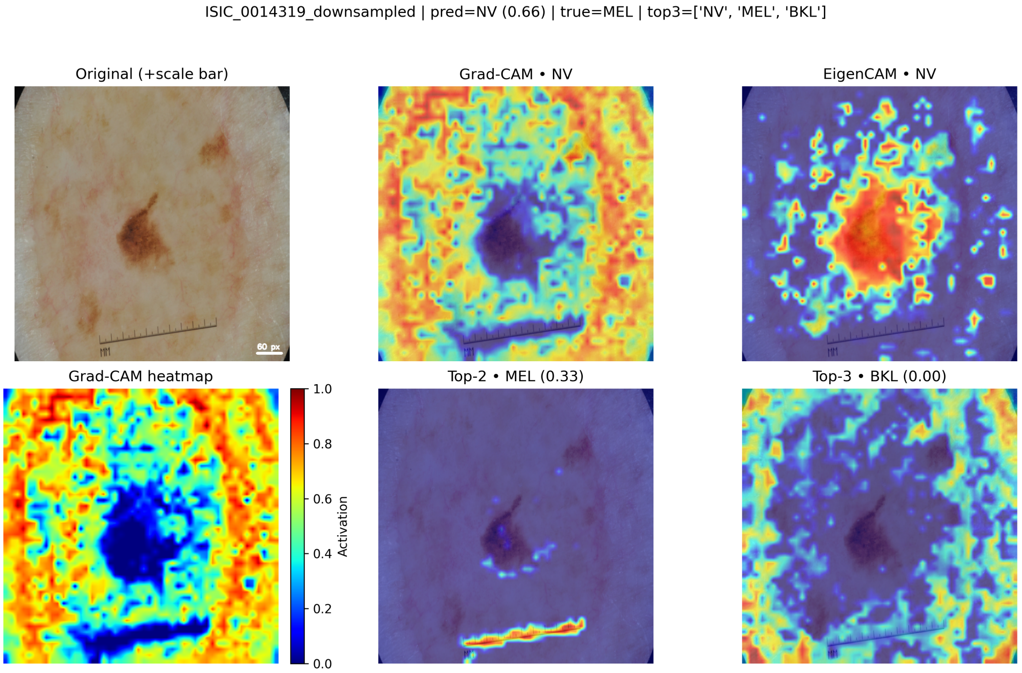

Figure S6. Grad-CAM and EigenCAM visualizations for a misclassified lesion by the EVA-02 baseline model (ground-truth MEL predicted as NV with a score of 0.66). Grad-CAM highlights substantial activation in the surrounding skin and along the scale marker, while the lesion center exhibits relatively low activation. EigenCAM shows a different activation pattern, with responses distributed around and within the lesion. These visualizations provide qualitative insights into the misclassification, with the activation in surrounding skin and near the scale marker suggesting that contextual regions may be reflected in the model response, although the visualizations do not establish the specific features responsible for the prediction.

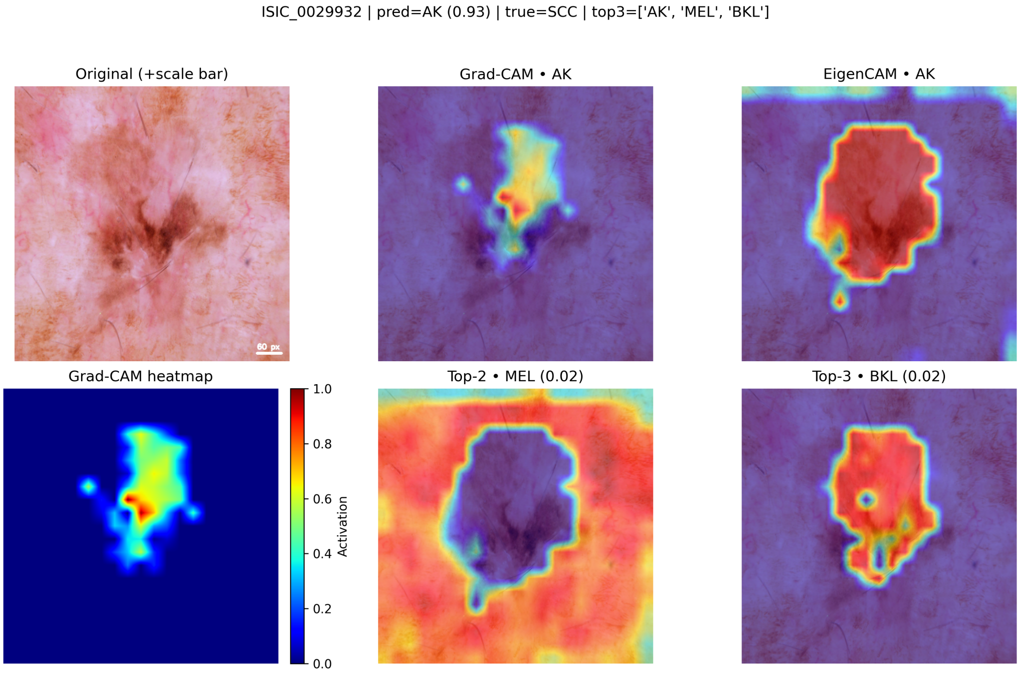

Figure S7. Grad-CAM and EigenCAM visualizations for a misclassified lesion by the CoAtNet baseline model (ground-truth SCC predicted as AK with a score of 0.93). Grad-CAM highlights a localized region near the center of the lesion, whereas EigenCAM shows broader activation across the lesion area.

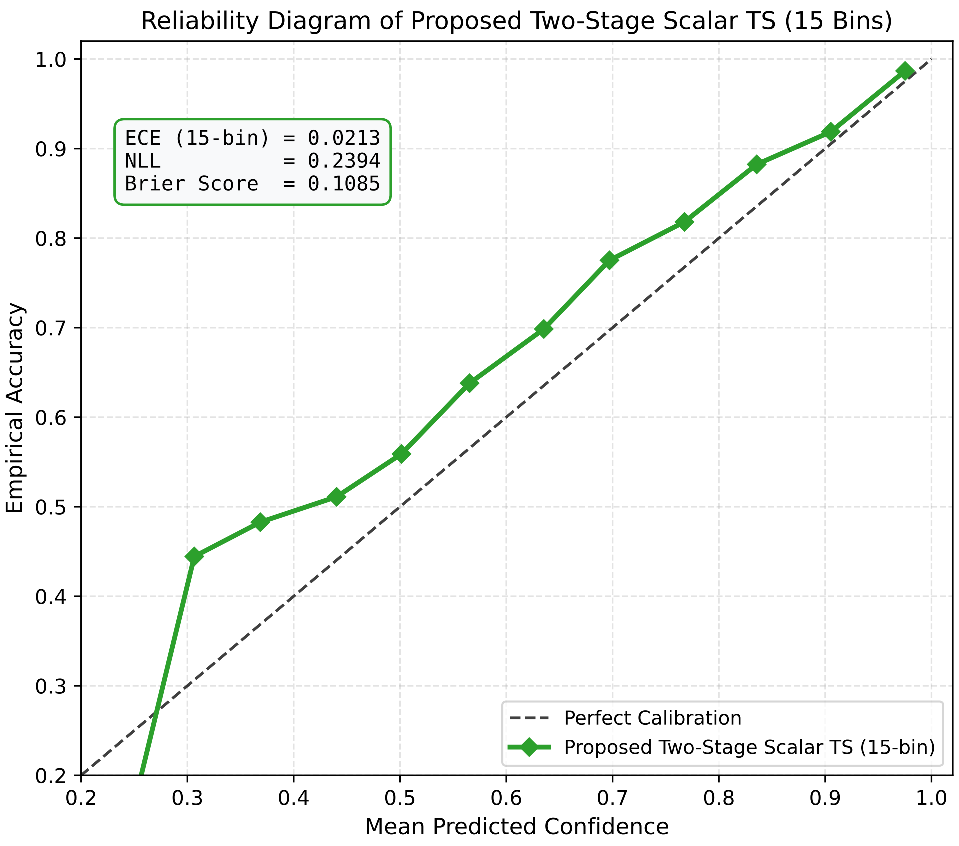

Figure S8. Calibration reliability diagram of the proposed meta-aware ensemble on the test set.The plot compares mean predicted confidence with observed accuracy across confidence bins. The curve closely follows the ideal diagonal reference, particularly in the medium and high confidence ranges, indicating well-aligned probability estimates. The Expected Calibration Error (ECE) is 0.0213, suggesting that the ensemble produces reliable and clinically interpretable confidence scores. A mild under-confidence is observed in low-probability regions, which is generally considered a conservative and acceptable behavior in safety-critical medical decision support systems.
