# Supplementary material for: Calibration-Aware Heterogeneous Multimodal Ensemble for Multi-Class Skin Lesion Diagnosis
Source: Diagnostics (Basel). 2026 Nov 1;16(21):3613. doi: 10.3390/diagnostics16213613 (PMC13762094; doi:10.3390/diagnostics16213613)
Supplement: Supplementary file 1 [file diagnostics-16-03613-s001.zip › supplementary-tex.zip!/Definitions/logo-mdpi-eps-converted-to.pdf]

MDPI
