# Supplementary material for: Calibration-Aware Heterogeneous Multimodal Ensemble for Multi-Class Skin Lesion Diagnosis
Source: Diagnostics (Basel). 2026 Nov 1;16(21):3613. doi: 10.3390/diagnostics16213613 (PMC13762094; doi:10.3390/diagnostics16213613)
Supplement: Supplementary file 1 [file diagnostics-16-03613-s001.zip › supplementary-tex.zip!/fig6.pdf]

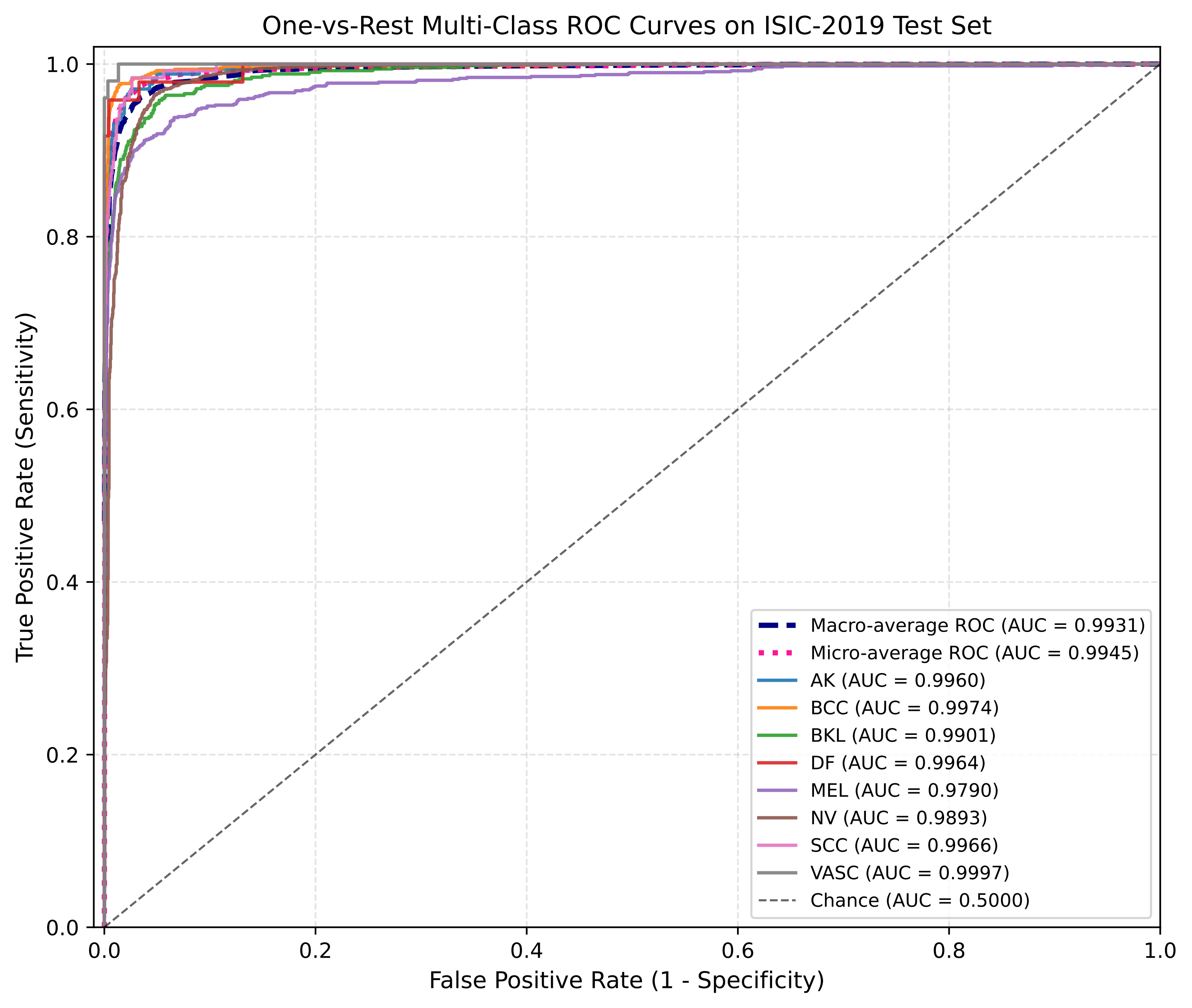
One-vs-Rest Multi-Class ROC Curves on ISIC-2019 Test Set
True Positive Rate (Sensitivity)
False Positive Rate (1 - Specificity)
0.0
0.2
0.4
0.6
0.8
1.0
Macro-average ROC (AUC = 0.9931)
Micro-average ROC (AUC = 0.9945)
AK (AUC = 0.9960)
BCC (AUC = 0.9974)
BKL (AUC = 0.9901)
DF (AUC = 0.9964)
MEL (AUC = 0.9790)
NV (AUC = 0.9893)
SCC (AUC = 0.9966)
VASC (AUC = 0.9997)
Chance (AUC = 0.5000)
