# Supplementary material for: Calibration-Aware Heterogeneous Multimodal Ensemble for Multi-Class Skin Lesion Diagnosis
Source: Diagnostics (Basel). 2026 Nov 1;16(21):3613. doi: 10.3390/diagnostics16213613 (PMC13762094; doi:10.3390/diagnostics16213613)
Supplement: Supplementary file 1 [file diagnostics-16-03613-s001.zip › supplementary-tex.zip!/fig7.pdf]

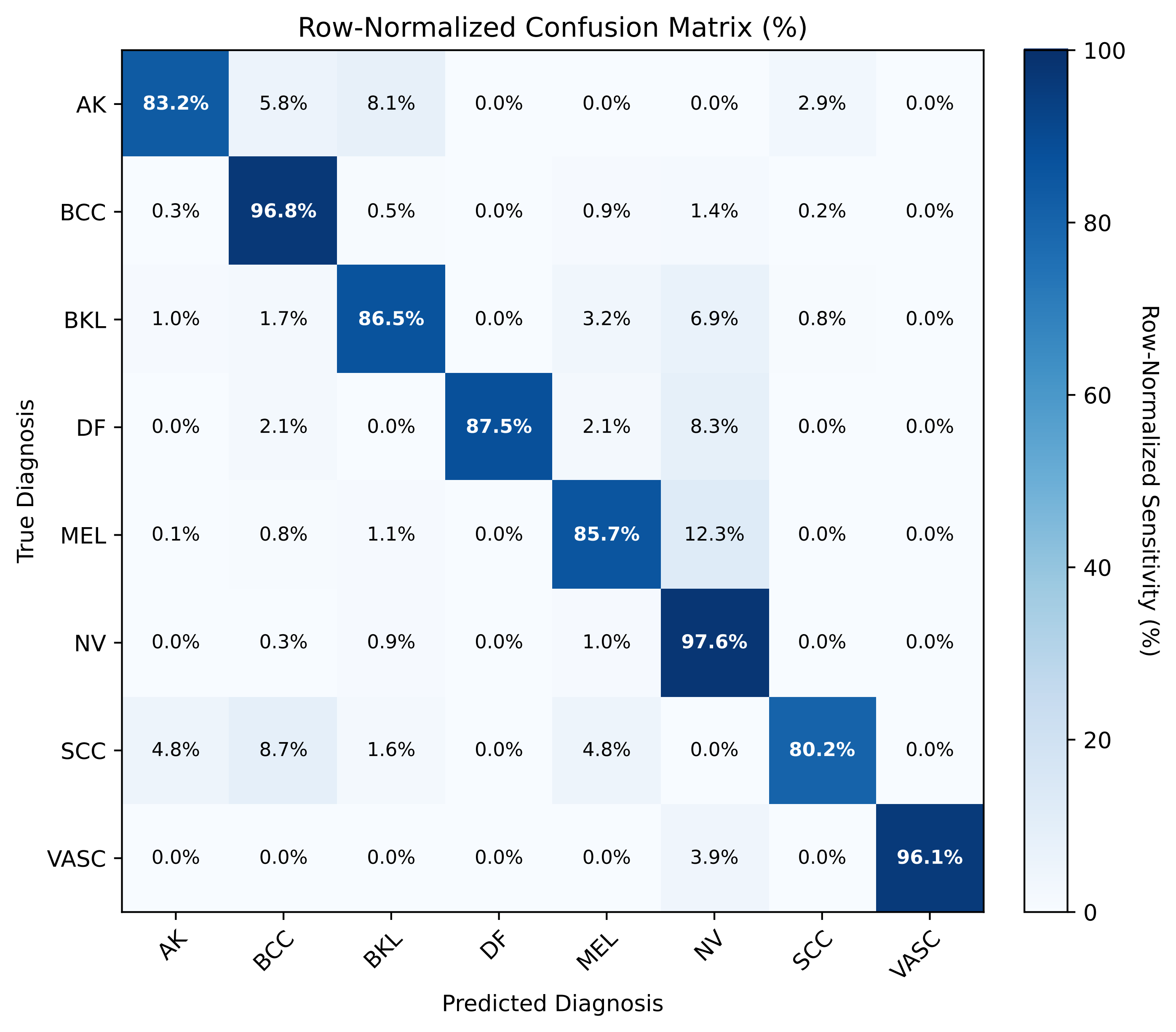

Row-Normalized Confusion Matrix (%)
True Diagnosis
Predicted Diagnosis
Row-Normalized Sensitivity (%)
0
20
40
60
80
100
AK
BCC
BKL
DF
MEL
NV
SCC
VASC
AK: 83.2% 5.8% 8.1% 0.0% 0.0% 0.0% 2.9% 0.0%
BCC: 0.3% 96.8% 0.5% 0.0% 0.9% 1.4% 0.2% 0.0%
BKL: 1.0% 1.7% 86.5% 0.0% 3.2% 6.9% 0.8% 0.0%
DF: 0.0% 2.1% 0.0% 87.5% 2.1% 8.3% 0.0% 0.0%
MEL: 0.1% 0.8% 1.1% 0.0% 85.7% 12.3% 0.0% 0.0%
NV: 0.0% 0.3% 0.9% 0.0% 1.0% 97.6% 0.0% 0.0%
SCC: 4.8% 8.7% 1.6% 0.0% 4.8% 0.0% 80.2% 0.0%
VASC: 0.0% 0.0% 0.0% 0.0% 0.0% 3.9% 0.0% 96.1%
