# Supplementary material for: Calibration-Aware Heterogeneous Multimodal Ensemble for Multi-Class Skin Lesion Diagnosis
Source: Diagnostics (Basel). 2026 Nov 1;16(21):3613. doi: 10.3390/diagnostics16213613 (PMC13762094; doi:10.3390/diagnostics16213613)
Supplement: Supplementary file 1 [file diagnostics-16-03613-s001.zip › supplementary-tex.zip!/flowchart.pdf]

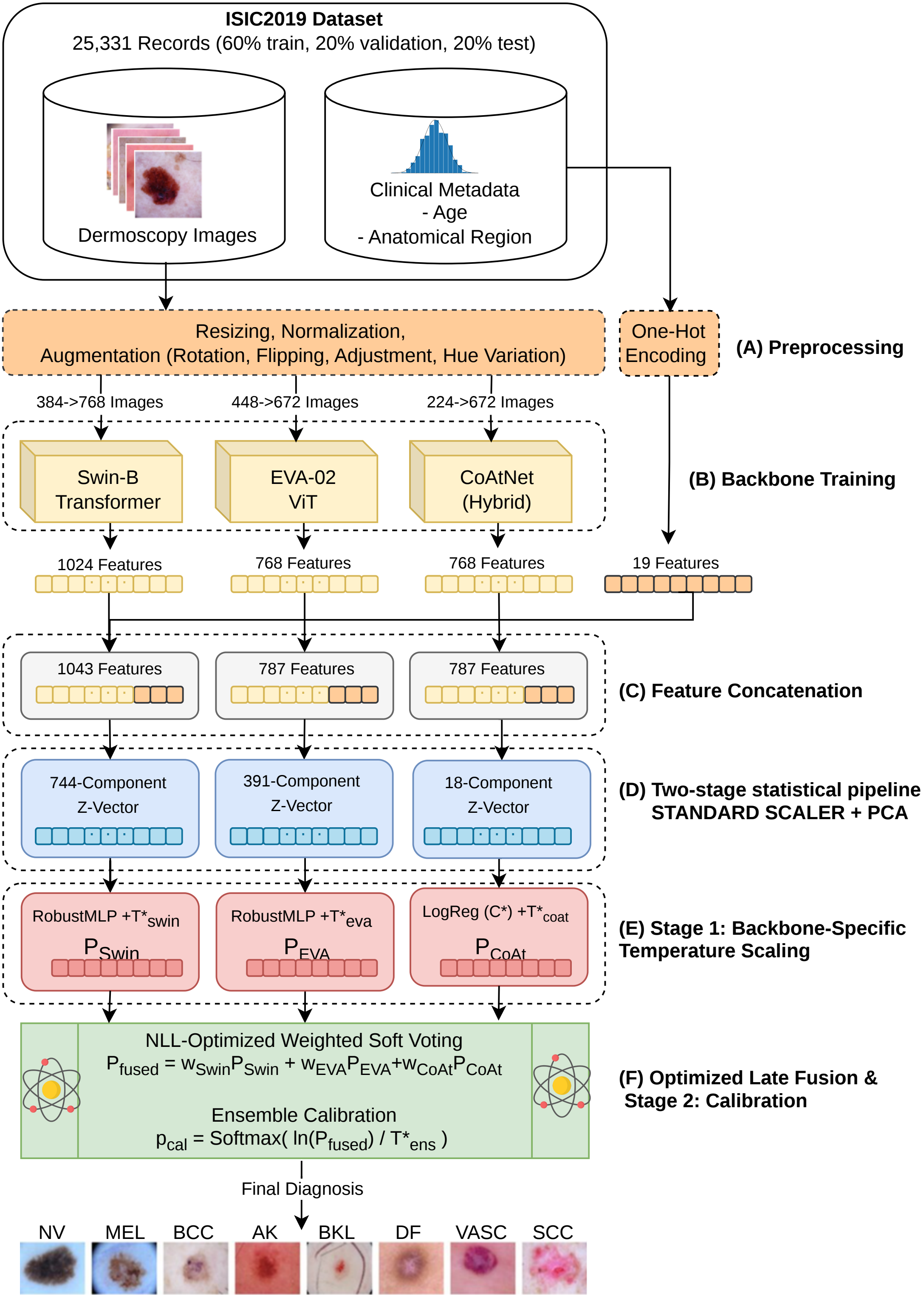

The StandardScaler and PCA are refitted on Train + Validation, with the PCA dimensionality and metadata mapping fixed. Classifier heads are retrained, while calibration temperatures and fusion weights remain fixed during final test evaluation.
