# Supplementary material for: Calibration-Aware Heterogeneous Multimodal Ensemble for Multi-Class Skin Lesion Diagnosis
Source: Diagnostics (Basel). 2026 Nov 1;16(21):3613. doi: 10.3390/diagnostics16213613 (PMC13762094; doi:10.3390/diagnostics16213613)
Supplement: Supplementary file 1 [file diagnostics-16-03613-s001.zip › supplementary-tex.zip!/Sup_Fig1.pdf]

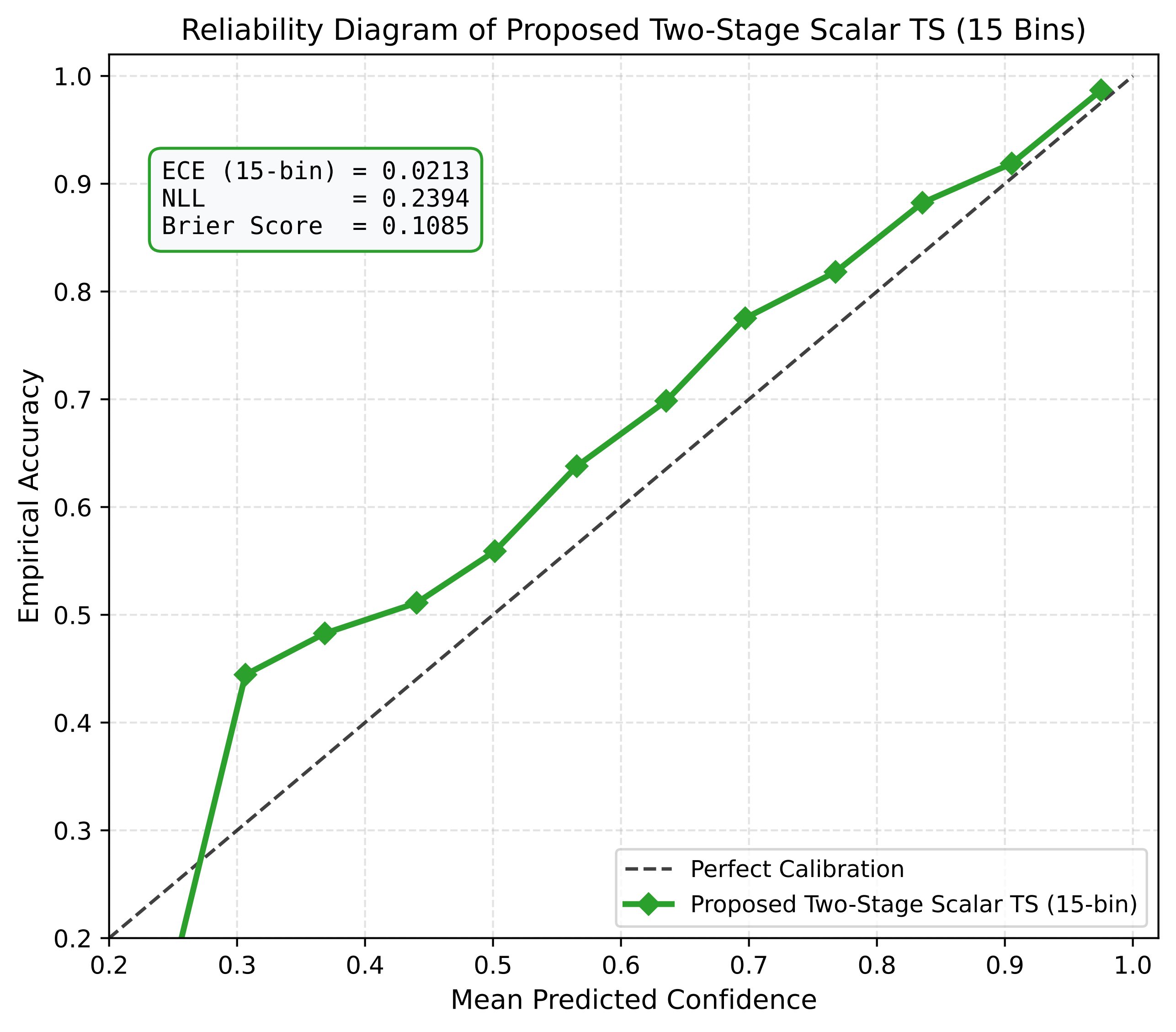
Reliability Diagram of Proposed Two-Stage Scalar TS (15 Bins)
ECE (15-bin) = 0.0213
NLL = 0.2394
Brier Score = 0.1085
Empirical Accuracy
Mean Predicted Confidence
0.2
0.3
0.4
0.5
0.6
0.7
0.8
0.9
1.0
Perfect Calibration
Proposed Two-Stage Scalar TS (15-bin)
